# Chromatin structures from integrated AI and polymer physics model

**DOI:** 10.1371/journal.pcbi.1012912

**Published:** 2025-04-09

**Authors:** Eric R. Schultz, Soren Kyhl, Rebecca Willett, Juan J. de Pablo

**Affiliations:** 1 Pritzker School of Molecular Engineering, The University of Chicago, Chicago, Illinois, United States of America; 2 Department of Statistics and Computer Science, The University of Chicago, Chicago, Illinois, United States of America; 3 Tandon School of Engineering, New York University, Brooklyn, New York, United States of America; 4 Materials Science Division, Argonne National Laboratory, Lemont, Illinois, United States of America; Virginia Tech, Computer Science and Physics, UNITEDSTATES OF AMERICA

## Abstract

The physical organization of the genome in three-dimensional space regulates many biological processes, including gene expression and cell differentiation. Three-dimensional characterization of genome structure is critical to understanding these biological processes. Direct experimental measurements of genome structure are challenging; computational models of chromatin structure are therefore necessary. We develop an approach that combines a particle-based chromatin polymer model, molecular simulation, and machine learning to efficiently and accurately estimate chromatin structure from *indirect* measures of genome structure. More specifically, we introduce a new approach where the interaction parameters of the polymer model are extracted from experimental Hi-C data using a graph neural network (GNN). We train the GNN on simulated data from the underlying polymer model, avoiding the need for large quantities of experimental data. The resulting approach accurately estimates chromatin structures across all chromosomes and across several experimental cell lines despite being trained almost exclusively on simulated data. The proposed approach can be viewed as a general framework for combining physical modeling with machine learning, and it could be extended to integrate additional biological data modalities. Ultimately, we achieve accurate and high-throughput estimations of chromatin structure from Hi-C data, which will be necessary as experimental methodologies, such as single-cell Hi-C, improve.

## Author summary

Eukaryotic chromosomes are carefully organized within the nucleus into three-dimensional structures. In different cells within the same organism, the genetic content of these chromosomes is identical, but the chromosome structures differ. This variation in chromosome structure can regulate gene expression and, therefore, control cell development and physiology. However, a detailed understanding of the relationship between genome organization and gene expression is still lacking. Developing computational models to explain this relationship is crucial to understanding the mechanisms controlling chromosome structure and predicting properties of gene expression from DNA structure. Here, we present an approach for inferring the 3D structure of chromatin from experimental Hi-C data using a physics-based model. By leveraging advances in machine learning in our modeling approach, we show that it is possible to predict chromatin structures accurately and efficiently. The high computational efficiency of the proposed approach will be essential for understanding structural variation in settings requiring high-throughput analysis, such as within populations of cells.

## Introduction

Advances in the ability to measure genome structure have gradually established that the three-dimensional organization of the genome is critical to the regulation of gene expression [1,2]. Existing experimental tools for characterizing genome structure fall into two fundamental categories: microscopy-based methods and sequencing-based methods. Microscopy methods can directly measure the 3D chromatin structures of individual cells by using fluorescence in situ hybridization (FISH) tags to target specific genomic loci [3,4]. Currently, technical challenges limit these experiments to measuring thousands of loci per experiment [4]. As a consequence, these experiments can either image a small genomic region at high resolution or a large region at low resolution, but not both.

On the other hand, sequencing technologies, such as Hi-C, can measure genome-wide chromatin organization at very high resolution, albeit indirectly [5,6]. Hi-C quantifies three-dimensional chromatin organization by measuring the contact frequency between genomic regions. The resulting contact matrix (also referred to as a contact map) contains the population-averaged contact frequency between pairs of genomic loci. Patterns in Hi-C data provide evidence for genome structural properties such as loops [6,7], topologically-associated domains (TADs) [8,9], and compartmentalization [5,10]. However, while Hi-C can measure contact frequencies at high resolution, estimating the three-dimensional structure of chromatin from Hi-C data has been a major challenge limiting the utility of Hi-C methods.

Computational models of chromatin structure provide a useful tool for elucidating the full three-dimensional structures of chromatin from Hi-C data [11,12]. We rely on particle-based polymer models. Such models of chromatin have been shown to reproduce many properties of its structure [13–16]. In these models, each particle typically represents between 5 kb and 1 Mb of DNA [12]. At this coarse of resolution, parametrization by traditional ‘bottom-up’ coarse-graining of atomic-scale or nucleosome-scale DNA models has not yet been accomplished [17,18]. Instead, these models have been parametrized ‘top-down’ by inferring thermodynamic parameters from a target experimental contact map [12].

Polymer modeling approaches in this framework can leverage the maximum entropy principle [19] to iteratively optimize thermodynamic parameters until the simulated chromatin ensemble best reproduces the experimental contact map [13,15,16] (reviewed in [11]). However, the iterative nature of this optimization procedure is a major computational bottleneck in the simulation procedure. Faster methods are essential for exploring variations in chromatin structure across cell types, analyzing structural diversity in single-cell variants of Hi-C data, assessing the effect of epigenetic factors on chromatin structure, and other large-scale investigations of variations in structure. As an alternative to the maximum entropy approach, analytical methods for estimating parameters from Hi-C data are an emerging research area [20,21]. While existing approaches are efficient, they rely on a reference homopolymer system, which limits their efficacy. Standard homopolymer potentials do not reproduce experimental chromatin contact probability scaling, which can limit the accuracy of the resulting structures.

Recent advances in machine learning methods, such as graph transformers [22], which combine graph neural networks [23] with attention mechanisms [24], present an opportunity for improvement. We show that adapting these advances in machine learning tools to the task of chromatin structure prediction can dramatically accelerate structure estimation from Hi-C data. Graph neural networks are a promising architecture because the Hi-C contact map can be considered as a weighted adjacency matrix of a graph. Some existing methods that treat Hi-C data as a graph include Higashi [25], a hypergraph neural network developed for imputation of sparse single-cell Hi-C data, and Sub-Compartment Identifier (SCI) [26], which uses a graph embedding step to predict chromatin sub-compartments from Hi-C data. Transformers are a class of neural networks that rely on attention mechanisms and have been popularized by their recent success in large language models [27,28]. Beyond language models, AlphaFold and its successor, AlphaFold 2, are graph transformers successful in the protein structure prediction problem [29–31]. We use a graph transformer with a unique approach that leverages state-of-the-art polymer models of chromatin structure (See Section S5 in S1 Appendix for further discussion).

Specifically, *we develop a graph neural network to predict the thermodynamic parameters of a polymer model from experimental Hi-C data, and these parameters allow us to simulate chromatin structures from the corresponding polymer model*. A key contribution of our approach is that we train the neural network exclusively on simulated data from the polymer model, circumventing the need for large quantities of high-quality experimental data. Further, we train our neural network to predict the interaction parameters of the polymer model rather than directly predicting a single structure. Compared to an approach that predicts only a single consensus structure, sampling structures via the polymer model better reflects both (a) uncertainty in structure estimates and (b) the dynamic nature of chromatin. Compared to using the iterative maximum entropy procedure to estimate the parameter of our polymer model, our GNN approach is 6.5x faster but yields structures of comparable quality.

## Results

### Overview of approach

**Fig 1:**
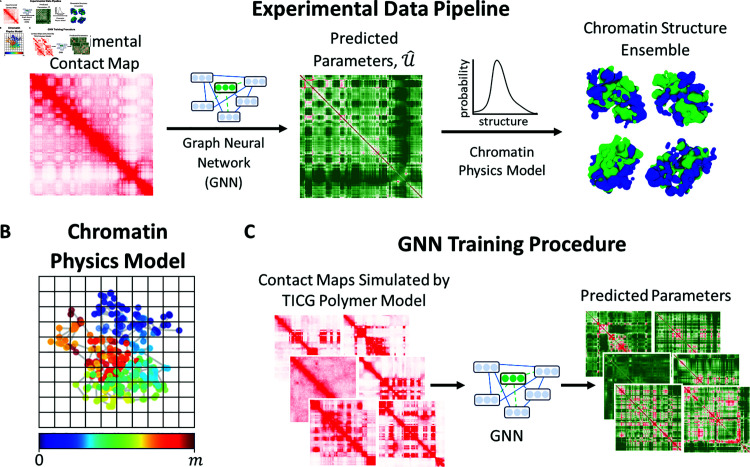
Overview of key elements. (A) Schematic overview of the experimental data pipeline. Given an experimental contact map, we construct a probability distribution over chromatin structures corresponding to our chromatin polymer model. The graph neural network (GNN) uses the contact map as an input and predicts interaction parameters, $\hat{U}$, of our chromatin physics model. Given these parameters, the polymer model samples chromatin structures at random. Structures are colored by A/B compartmentalization as determined by the sign of the first principal component of the experimental genomic-distance normalized contact map. (B) Schematic overview of GNN training procedure. We train the GNN on simulated contact maps generated by the chromatin physics model where we know the ground truth interaction parameters. (C) Schematic illustration of chromatin polymer model. Particles are colored according to their position along the polymer to aid visualization. Particles in the same grid cell are defined as in contact. Grid size is not to scale.

Broadly speaking, we aim to estimate the underlying chromatin structure from experimental Hi-C data (Fig 1A). We use a particle-based physics model where we model chromatin as a heteropolymer of *m* particles; each particle represents 50 kb of DNA (Fig 1B). Particles interact in our model in a pairwise fashion controlled by an interaction energy matrix, *U*, which contains an interaction energy, $U_{ij}$, for every pair of particles *i* and *j*. To determine *U*, we train a graph neural network (GNN) that inputs an experimental contact map and outputs the estimated interaction parameters, ${\hat{U}}^{\text{GNN}}$, of the model (Fig 1C). Given the estimated interaction parameters, we use our polymer model to generate a distribution of structures. If the generated structures are accurate, we will be able to calculate a simulated contact map from these structures (Eq (4)) that closely resembles the target experimental contact map.

Obtaining sufficient data to train the neural network is a major challenge, and a key contribution of our approach is that we are able to train the GNN on simulated data from our polymer model. Briefly, we first use the existing maximum entropy (ME) optimization to estimate ${\hat{U}}^{\text{ME}}$ for 33 experimental contact maps all from sub-regions of the odd chromosomes from the IMR90 cell line. Then, we draw synthetic polymer model parameters from probability distributions fitted to these parameter estimates. Finally, we conduct simulations using our polymer model and the synthetic parameters to yield realistic simulated contact maps. Using this dataset, we train the GNN to predict the synthetic polymer model parameters. Intuitively, the GNN is learning to estimate the thermodynamic parameters that will yield a structural ensemble that best reproduces the experimental contact map.

After training the GNN, we can input an arbitrary 50 kb resolution experimental contact map and use the parameters, ${\hat{U}}^{\text{GNN}}$, predicted by the GNN to simulate the corresponding chromatin ensemble (Fig 1). We will contrast this approach for simulating chromatin structures (GNN approach) with using the maximum entropy optimization to estimate the simulation parameters (maximum entropy approach). Pseudocode for both of these approaches can be found in S1 Appendix; maximum entropy approach: Algorithm D; GNN approach: Algorithm E.

### Performance on experimental Hi-C data

To demonstrate the accuracy and efficiency of our GNN approach, we model the chromatin structures corresponding to 40 experimental contact maps from a test set comprising the even chromosomes of the IMR90 cell line. For each experimental contact map, we use both the GNN and the maximum entropy approach to estimate the parameters of our polymer model. We compare the resulting simulated contact maps and the total simulation time of each method. We find that polymer simulations using the parameters estimated by the GNN accurately reproduce experimental contact maps despite requiring an order of magnitude less simulation time than the maximum entropy approach.

**Fig 2:**
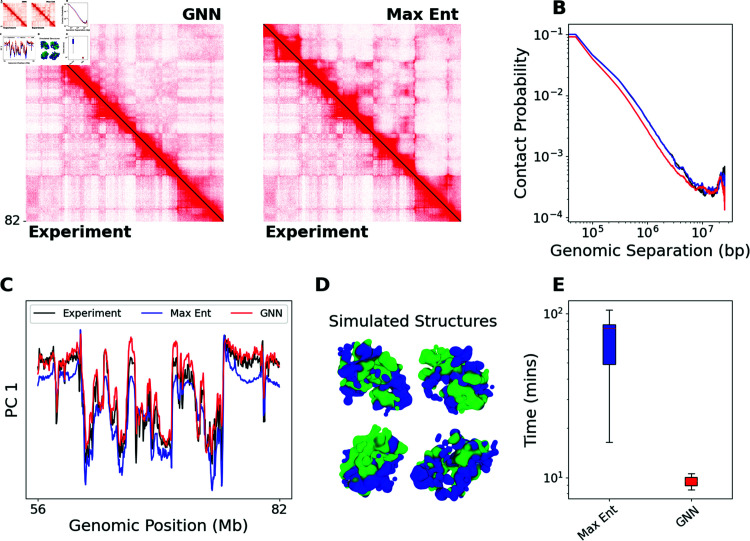
Chromatin simulation using GNN approach accurately reproduces experimental contact map. (A) Contact maps of IMR90 Chr2:56.3-81.9 Mb. The lower triangle shows the experimental contact map. The color bar is the same for both subfigures. *Left:* The upper triangle shows the GNN simulated contact map. *Right:* The upper triangle shows the maximum entropy simulated contact map. (B–C) Comparison of the experiment (black), maximum entropy simulation (blue), and GNN simulation (red). (B) Comparison of contact probability scaling as a function of genomic separation on a log-log scale. (C) Comparison of first principal component of genomic-distance normalized contact maps. (D) A representative structure from GNN simulation. Colored by A/B compartmentalization. (E) Comparison of total run time in minutes for all contact maps in the experimental test set  ( *n* = 40 ) . Boxes represent the 25th and 75th quantiles, and the solid line indicates the median. Whiskers extend from the box by 1.5 times the interquartile range.

An example result for a 25.6 Mb region of GM12878 Chr2 is shown in Fig 2. The simulated contact maps closely resemble the experimental contact map visually (Fig 2A). Both simulations clearly reproduce the A/B compartmentalization [5] of the experimental contact map. The A/B compartmentalization can be seen visually as the plaid pattern in the Hi-C contact map, and is captured in the first principal component of the genomic-distance normalized contact map (Eq (5), Fig 2C). The contact probability scaling as a function of genomic distance reproduces the experimental scaling (Fig 2B). An example structure from the GNN simulation is shown in Fig 2D. The structure is colored according to the A/B compartmentalization as measured by the sign of the first principal component of the genomic-distance normalized contact map. The A compartment (green) and B compartment (blue) are seen to separate in three-dimensional space. The simulated contact maps are computed from 30,000 such structures (Eq (4)).

Notably, the GNN approach is 6.5 times faster than the maximum entropy approach on average (Fig 2E). We report the average simulation time over an experimental test set of 40 contact maps from the even chromosomes of the IMR90 cell line. The reported time only considers the time required to sample structures with polymer model (Algorithm A in S1 Appendix) as the time of any other step is negligible in comparison. For the maximum entropy approach, the reported time includes all simulation time required for the maximum entropy optimization to converge, as well as a final simulation with the converged parameters (lines 6 and 11 in Algorithm D in S1 Appendix). The GNN approach circumvents the maximum entropy optimization entirely. Therefore, the time reported for the GNN approach is only the simulation time using the interaction parameters estimated by the GNN (line 2 in Algorithm E in S1 Appendix). The average time for simulations using the GNN approach is 10.3  ±  0.2 minutes. The maximum entropy method has a larger average simulation time of 66.5  ±  4.0 minutes.

In Table 1, we show include quantitative results averaged over the 40 contact maps from the even chromosomes of the IMR90 cell line. Since the maximum entropy approach depends on an optimization procedure, we include two definitions of convergence in Table 1. The maximum entropy optimization is terminated based on a convergence criterion of $\epsilon =10^{-2}$ or $\epsilon =10^{-3}$ (see Section S2.2.5 in S1 Appendix for details). Since the two convergence criteria yield comparably accurate simulated contact maps, we restrict the text to the results from $\epsilon =10^{-2}$, which is faster. Note that the maximum entropy approach is not guaranteed to converge. We find that all optimizations converge for $\epsilon =10^{-2}$ and 39 out of 40 converge for $\epsilon =10^{-3}$. Further tuning of the user-defined parameters of the maximum entropy optimization and of the number of structures sampled via the polymer model can improve the convergence success rate.

**Table 1:** Average results for 40 experimental contact maps from even chromosomes of IMR90. The maximum entropy optimization uses a convergence criterion of either $\epsilon =10^{-2}$ or $\epsilon =10^{-3}$. SCC is the stratum-adjusted correlation coefficient [32]. HiC-Spector is the HiC-Spector metric from [33]. Corr PC1($\tilde{H}$) is the Pearson correlation between the first principal component of the genomic-distance normalized contact maps, $\tilde{H}$. RMSE($\tilde{H}$) is the root-mean-squared error between the genomic-distance normalized contact maps. Simulation Time is each method’s total simulation time in minutes. See text for details. All values are mean  ±  standard error.

Method	SCC	HiC-Spector	Corr PC1($\tilde{H}$)	RMSE($\tilde{H}$)	Simulation Time
GNN	0.72 ± 0.01	0.51 ± 0.02	0.90 ± 0.01	0.65 ± 0.01	10.3 ± 0.2
Maximum Entropy (*ϵ*=1e-2)	0.81 ± 0.01	0.49 ± 0.02	0.78 ± 0.03	1.04 ± 0.03	66.5 ± 4.0
Maximum Entropy (*ϵ*=1e-3)*	0.84 ± 0.01	0.51 ± 0.02	0.80 ± 0.03	0.72 ± 0.02	212.4 ± 13.9

*1 out of 40 maximum entropy simulations failed to meet the convergence criterion of $\epsilon =10^{-3}$; we show results for the remaining 39.

We compare contact maps using two metrics from the Hi-C literature (Table 1) [32,33]. See Section S7 in S1 Appendix for further discussion of these metrics and for implementation details. The first metric is the stratum-adjusted correlation coefficient (SCC) [32]. The SCC is a weighted average of the Pearson correlations between pairs of off-diagonals of two contact maps. The simulated contact maps from simulations using the GNN approach have an SCC of 0.72  ±  0.01 (mean  ±  standard error) compared to an SCC of 0.81  ±  0.01 for the maximum entropy approach (Fig 2D). While the SCC for the GNN approach is lower than the maximum entropy approach, it is still higher than the SCC between experimental contact maps from different cell lines. The SCC between IMR90 and GM12878 contact maps of the same genomic regions is 0.58  ±  0.02. This suggests that the GNN is capturing cell line specific patterns of genome structure. The SCC requires two user-defined parameters; we explore the effect of these parameters in Section S7 and Fig H in S1 Appendix.

The second metric is the HiC-Spector score [33]. HiC-Spector compares contact maps by treating them as graph Laplacian matrices. It computes the sum of Euclidean norms between the top *κ* Laplacian eigenvectors of the two Hi-C contact maps. The metric is rescaled to the range [0,1], where higher is better. The GNN approach has a HiC-Spector scores of 0.51  ±  0.02 compared to 0.49  ±  0.02 for the maximum entropy approaches, which is not statistically significant by two-sided paired t-test.

We also include two simpler metrics, which both directly measure the similarity of genomic-distance normalized contact maps, $\tilde{H}$. It is common to visualize the first principal component of $\tilde{H}$, PC1($\tilde{H}$), as a measure of A/B compartmentalization. For this reason, we compute the Pearson correlation between PC1(${\tilde{H}}^{\text{sim}}$) and PC1(${\tilde{H}}^{\text{exp}}$) (Corr PC1($\tilde{H}$) in Table 1). The GNN approach has a mean Pearson correlation of 0.90  ±  0.01 between the first principal component of the simulated and experimental genomic-distance normalized contact maps. Meanwhile, the maximum entropy approach has a mean correlation of 0.78  ±  0.03. Finally, we directly measure the root-mean-squared error between genomic-distance normalized contact maps. The GNN approach has an RMSE($\tilde{H}$) of 0.65  ±  0.01 (vs 1.04  ±  0.03 for maximum entropy).

We observe that the GNN approach performs better on the metrics computed from the genomic-distance normalized contact maps, $\tilde{H}$: RMSE($\tilde{H}$) and Corr PC1($\tilde{H}$). On the other hand, the maximum entropy approach performs better on the metrics computed from the raw contact maps, *H*: SCC and HiC-Spector. We discuss the possible origins and implications of this difference in Section S7 in S1 Appendix.

### Generalization to other human and mouse cell lines

For all results in this manuscript, we use a GNN trained on simulated data that was generated based on a single experimental Hi-C dataset of the IMR90 human cell line. To demonstrate that the GNN can generalize to other cell lines beyond IMR90, we use the GNN approach to simulate experimental contact maps corresponding to the even chromosomes of the human GM12878, HMEC, HUVEC, and HAP1 human cell lines. Further, to assess generalization to other organisms, we include the CH12.LX mouse cell line. Despite being trained exclusively on simulated data based on the IMR90 cell line, the simulated contact maps from the GNN approach reproduce experimental contact maps from these cell lines.

**Fig 3:**
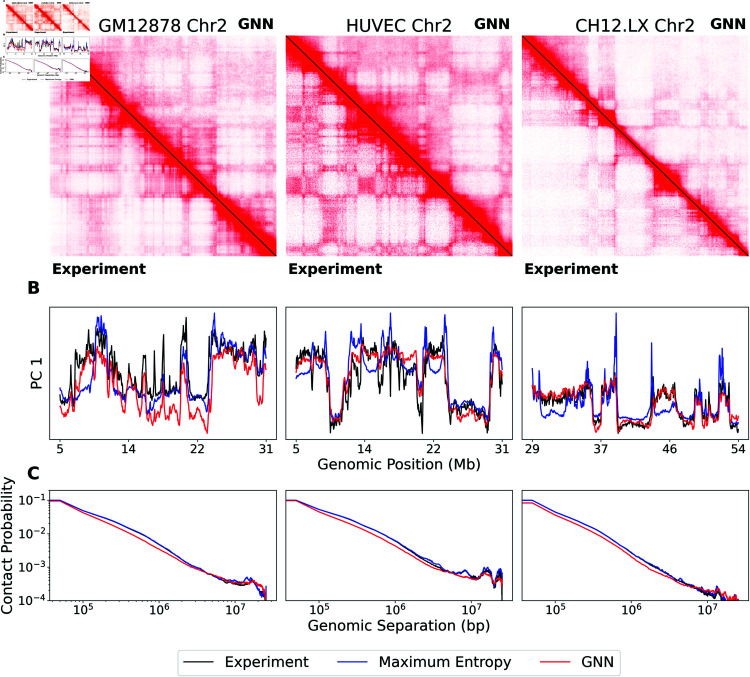
GNN generalizes to unseen cell types. *Left:* GM12878 human lymphoblastoid cell line Chr2:5.1-30.7 Mb. *Middle:* HUVEC murine umbilical vein endothelial cell line Chr2:5.1-30.7 Mb. *Right:* CH12.LX mouse lymphoma cell line. (A) Comparison of experimental and GNN simulated contact maps. The lower triangle shows the experiment, and the upper triangle shows the GNN simulation. The color bar is the same for all subfigures. SCCs between simulated and experimental contact maps are 0.67, 0.70, and 0.76, respectively. (B–C) Comparison of the experiment (black), maximum entropy (blue), and GNN (red). (B) Comparison of the first principal component of genomic-distance normalized contact maps. (C) Comparison of contact probability scaling.

In Fig 3A, we show example contact maps for the GM12878, HUVEC, and CH12.LX cell lines. The simulated contact maps reproduce A/B compartmentalization (Fig 3B) and contact probability scaling (Fig 3C). In Fig B in S1 Appendix, we show the simulated contact maps from the maximum entropy approach for comparison.

Averaged over 158 simulated contact maps from the four human cell lines, the results are comparable to those seen on the IMR90 cell line (Table B in S1 Appendix). The GNN approach had an SCC of 0 . 71 ± 0 . 01. In comparison, the maximum entropy approach had an SCC of 0 . 82  ±  0 . 00. On all other metrics, the GNN approach outperforms the maximum entropy approach: HiC-Spector Score (0 . 54 ± 0 . 01 vs 0 . 52 ± 0 . 01); Corr PC1($\tilde{H}$) (0 . 85 ± 0 . 01 vs 0 . 79 ± 0 . 01); and RMSE($\tilde{H}$) (0 . 70 ± 0 . 01 vs 1 . 07 ± 0 . 01). Further, the GNN approach was 6.6x faster on average.

The results for the mouse CH12.LX cell line are comparable to the results for the human cell lines. The GNN approach had an SCC of 0 . 78 ± 0 . 01 compared to 0 . 81 ± 0 . 01 for the maximum entropy approach, which is a smaller difference in SCC than for any of the human cell lines. The GNN approach outperforms the maximum entropy on all of the other metrics except for Corr PC1($\tilde{H}$) (0 . 80 ± 0 . 04 vs 0 . 85 ± 0 . 01) (Table B in S1 Appendix). For the mouse CH12.LX cell line, the GNN approach was 8.3x faster on average.

Table B in S1 Appendix includes results for each cell line separately. We do not see substantial variation in performance between cell lines. These results demonstrate that the GNN has robustly learned properties of Hi-C data that generalize across cell lines.

### Validation with super-resolution imaging

To directly validate the structures estimated from simulations using GNN-estimated parameters, we compare our simulated structures with experimental imaging data. Given an experimental contact map from the IRM90 cell line, we use the GNN to estimate the parameters of our polymer model, and we compare the estimated structures to super-resolution chromatin tracing experiments of IMR90 Chr2 [4] and Chr21 [3,4].

**Fig 4:**
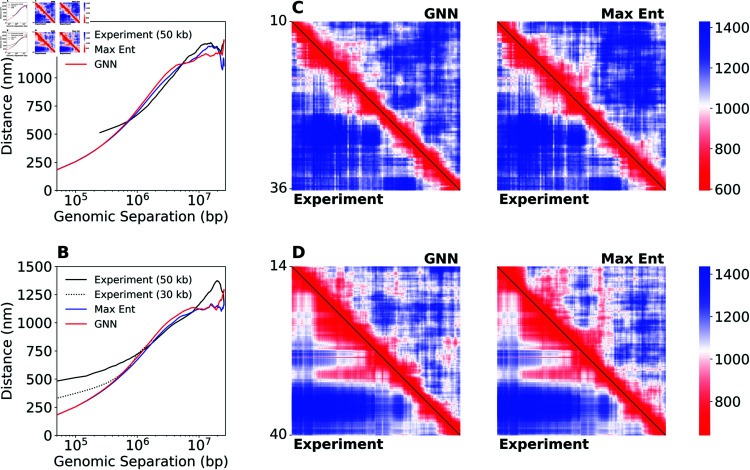
GNN reproduces patterns in spatial distances from super-resolution chromatin tracing experiments. We obtain experimental structures at 50 kb resolution Su et al. [3,4]. *Top row*: IMR90 Chr2:10-25.6 Mb. *Bottom row*: IMR90 Chr21:14-39.6 Mb. (**A–B**) Mean spatial distance scaling as a function of genomic separation for experiment (black), GNN simulation (red), and maximum entropy simulation (blue). (**B**) The additional dotted black line corresponds to an experimental structure of IMR90 Chr21:28Mb-29.2 Mb at 30 kb resolution from Bintu et al. [3]. (**C–D**) Mean spatial distance matrices. The lower triangle shows the experimental distance matrix. *Left:* The upper triangle shows the GNN simulated distance matrix. *Right:* The upper triangle shows the maximum entropy simulated distance matrix.

We note that the energy functional of our simulation does not directly constrain distances, nor is the GNN trained to reproduce distances. The two parameters in our model that most directly affect distances are the bond length, *b*, between neighboring particles and the simulation volume, *V*. We choose a bond length (*b* = 200nm) and simulation volume (*V* = 8 *μ*m^3^) such that the mean distance scaling as a function of genomic separation is comparable between the simulation and experiment (Fig 4AB and Section S2.1.5, Fig D in S1 Appendix).

The simulated structures are in good agreement with the experimental structures. On chromosome 2, the simulated and experimental mean distance matrices have a Pearson correlation of 0.89 for the GNN (maximum entropy: 0.90) (Fig 4C). On chromosome 21, the simulated and experimental mean distance matrices have a Pearson correlation of 0.78 for the GNN (maximum entropy: 0.83) (Fig 4D). These results help suggest that the simulated structures are representative of the underlying experimental structures. The corresponding simulated contact maps are included in Fig C in S1 Appendix.

The accuracy of the simulated distances depends greatly on the choice of bonded parameters and bonded potential term. A more accurate bonded term in the energy functional could improve these results [34]. Importantly, the distance matrices from the GNN approach are comparable to the maximum entropy approach, as both methods use the same bonded term. Improving the underlying polymer model would improve both results.

## Discussion

To improve our understanding of genome organization and gene expression, characterizing chromatin structure is essential. With efficient approaches for modeling chromatin structure from Hi-C data, we can compare chromatin structures across many cells and cell types. These chromatin models can unveil the regulatory mechanisms behind gene expression to answer questions about cell differentiation and development.

We introduce an efficient computational pipeline for estimating chromatin structures from Hi-C data. Our approach combines advances in polymer modeling of chromatin structure with modern machine learning methods to predict chromatin structures significantly faster than classical approaches. A key contribution of our approach is that we train the GNN exclusively on simulated data. This was made possible by leveraging the polymer model to generate large quantities of simulated contact maps. Further, because we train the GNN to predict thermodynamic parameters of the polymer model instead of predicting structures, the GNN does not need to learn the physics of chromatin folding. We believe this choice makes the GNN more robust to variation in genome structure across different cell lines as well as simplifies training. One obvious drawback of this choice is that we cannot overcome any limitations of the physics in our polymer model. As biophysical modeling of chromatin improves, we can retrain the GNN using more sophisticated chromatin models.

Using our GNN approach is an order of magnitude faster than using the maximum entropy approach to estimate the parameters of our polymer model. At the same time, the accuracy of the predicted chromatin structures is comparable to structures from simulations using parameters estimated with the maximum entropy approach. In addition, the GNN does not require *a priori* knowledge or assumptions about the particle labels, *Ψ*, of the chromatin model, whereas the maximum entropy approach requires the particle labels as an input. Notably, we show that despite training the GNN exclusively on cells from one human cell line (IMR90) it is still able to generalize to other human and even mouse cell lines.

Our approach has the potential to be useful in the analysis of single-cell variants of Hi-C [35]. These single-cell Hi-C variants can generate tens of thousands of single-cell contact maps, providing an opportunity to compare chromatin structures across cells and cell populations [36]. If using the existing maximum entropy approach, it would be incredibly computationally expensive to simulate the structure of each single-cell contact map. One challenge is the sparsity of single-cell Hi-C data poses a problem for both the GNN and maximum entropy approaches. (see Section S4, Fig F in S1 Appendix). Future work will address how to generalize our graph neural network to the single-cell setting, potentially by leveraging existing imputation procedures such as scHiCluster [37] or Higashi [25] to infer missing contacts in single-cell Hi-C data *in silico*.

Our GNN approach can be extended to consider additional biological data modalities. Future work can explore adapting our approach to predict structures from chromatin microscopy data instead of Hi-C. Alternatively, our approach could be extended to account for settings where multiple data modalities are present - such as Hi-C data in addition to image-based data. In principle, a machine learning-based approach could be robust to the presence/absence of multiple data modalities.

More broadly, our approach can be considered a general framework for combining physics-based modeling with machine learning (Section S6 in S1 Appendix). Machine learning approaches such as machine-learned force fields (MLFFs) have become prevalent in ‘bottom-up’ modeling [38], where a coarse-grained model is used to approximate an existing fine-resolution model. On the other hand, our GNN approach falls in the category of ‘top-down’ modeling, which seeks to predict model parameters from experimental data [39]. There have been limited examples of machine learning methods developed for ‘top-down’ modeling [40–43]. Future work could extend our approach to other ‘top-down’ settings where physics-based models are inferred from data.

## Materials and methods

### Experimental data

We obtain experimental Hi-C contact maps, *H*, at 50 kb resolution. Experimental data files are downloaded from Juicebox [44] or ENCODE [45–48], as indicated in Table C in S1 Appendix. Contact maps are downloaded as raw counts without any pre-processing. We show that our approach performs comparably when using contact maps pre-processed with ICE [49] or Knight-Ruiz [50] matrix balancing in (Section S3, Fig E in S1 Appendix). We include only autosomal chromosomes. We divide chromosomal contact maps into 25.6 Mb regions, corresponding to *m* = 512 bins per contact map. We only choose regions such that $H_{ii}>0$ for all *i*. Doing so avoids centromeric and telomeric regions as well as regions with low read-mappability. We normalize the contact maps such that the average contact probability between beads *i* and beads *i*  ±  1 is 0.1. We then overwrite the main diagonal (self-self interactions) with 1’s.

### Particle-based chromatin model

We model chromatin as a heteropolymer of *m* particles, where each particle represents 50 kb of DNA. The potential energy function of our polymer model can be decomposed into bonded, $U^{\text{b}}(\vec{r})$, and nonbonded, $U^{\text{nb}}(\vec{r})$, terms:


$$\begin{array}{ccc} U(\vec{r})=U^{\text{b}}(\vec{r})+U^{\text{nb}}(\vec{r}) & & \end{array}$$ (1)

where $\vec{r}\in {\mathbb{R}}^{m\times 3}$ contains the positions of all *m* particles in the simulation and $U^{\text{b}}(\vec{r})$ is a Gaussian chain bonded potential (Section S2.1 in S1 Appendix).

The nonbonded term is derived from the Theoretically Informed Coarse Grained (TICG) potential for block copolymers [51–54]. While there are several existing chromatin models in the literature [13,15,16,55], we choose the TICG potential because of the computational efficiency resulting from its grid-based implementation (Fig 1B]. Instead of computing the spatial distance between particles, the TICG potential defines particles located in the same grid cell to be interacting ($I({\vec{r}}_{i},{\vec{r}}_{j})=1$). Omitting some constants, the nonbonded potential can be written as follows:


$$\begin{array}{ccc} U^{\text{nb}}(\vec{r})=\underset{i,j\in \{1,\ldots ,m\}}{\sum }I({\vec{r}}_{i},{\vec{r}}_{j})U_{ij} & & \end{array}$$ (2)

where ${\vec{r}}_{i}$ denotes the position of particle *i*, $I({\vec{r}}_{i},{\vec{r}}_{j})\in \{0,1\}$ indicates if particles *i* and *j* are in same grid cell, and $U\in {\mathbb{R}}^{m\times m}$ is a matrix of interaction parameters between pairs of particles.

The nonbonded potential can be further decomposed as:


$$\begin{array}{ccc} U^{\text{nb}}(\vec{r})=\underset{i,j\in \{1,\ldots ,m\}}{\sum }I({\vec{r}}_{i},{\vec{r}}_{j})(L_{ij}+T_{ij}) & & \end{array}$$ (3)

where $L,T\in {\mathbb{R}}^{m\times m}$ are two types of interaction parameters and *U* : = *L* + *T*.

Note that the original description of the TICG potential is written in terms of local densities of particles within each grid cell rather than pairs of particles [52]. In Section S2.1 in S1 Appendix, we include a description in the local density notation and a derivation of Eq (2) and Eq (3). We will use the notation in Eq (2) and Eq (3) for both the maximum entropy and GNN approaches.

Here, we will provide some intuition for the role of *L* and *T*. Both terms are analogous to terms in the MiChrom model proposed by di Pierro *et al.* [13]. *L* is low-rank by construction and controls interactions between particles of different epigenetic states. The *L* contribution is a generalization of the “chromatin types” term used in the MiChrom model. *T* is a symmetric matrix where all descending diagonals are constant-valued (i.e., a Toeplitz matrix). As a result, all particles with the same genomic distance, *d* = | *i*  −  *j* | , will have the same value $T_{ij}$. *T* serves to modify the scaling behavior of the polymer chain to match the experimental contact probability scaling. The *T* contribution is analogous to the “ideal chromosome” term used in the MiChrom model [13]. We define $U_{ij}:=L_{ij}$  +  $T_{ij}$ as a net pairwise interaction strength. Intuitively, *U* can be considered a look-up table for the interaction energy between every pair of particles *i* and *j*. Pairs of particles with negative $U_{ij}$ will attract, while those with positive $U_{ij}$ will repel.

Given *U*, we simulate chromatin structures by Monte Carlo sampling (Section S2.1.6 in S1 Appendix). With these structures, we can calculate any desired structural property. One property of particular interest is the simulated contact map, $H^{\text{sim}}$, which we use to assess the quality of our simulation. We define $H^{\text{sim}}$ as the ensemble average (i.e., averaged over all structures) of the grid indicator function, $I({\vec{r}}_{i},{\vec{r}}_{j})$:


$$\begin{array}{ccc} H_{ij}^{\text{sim}}={<I({\vec{r}}_{i},{\vec{r}}_{j})>}_{\vec{r}} & & \end{array}$$ (4)

We typically estimate $H^{\text{sim}}$ by averaging over 30,000 structures sampled via the polymer model. We expect the simulated contact map, $H^{\text{sim}}$, to accurately reproduce the experimental contact map, *H*.

### Data generation

We obtain a set of experimental contact maps, $H^{\left(\ell \right)}$ for *ℓ* = 1 , … , 33 corresponding to 25.6 Mb regions at 50 kb resolution of the odd chromosomes of the IMR90 cell line. Each contact map contains *m* = 512 bins (i.e., rows/cols). For each $H^{\left(\ell \right)}$, we use the maximum entropy approach to estimate ${\hat{L}}^{\text{ME}(\ell )}$ and ${\hat{T}}^{\text{ME}(\ell )}$ matrices from Eq (3). Define ${\hat{U}}^{\text{ME}(\ell )}:={\hat{L}}^{\text{ME}(\ell )}$  +  ${\hat{T}}^{\text{ME}(\ell )}$. See Section 2.2 in S1 Appendix for a complete description of the maximum entropy optimization.

We use the following procedure to generate 10,000 synthetic interaction parameter matrices, $U^{\left(s\right)}\in R^{m\times m}$ for *s* = 1 , … , 10000:

1. Generate random $L^{\left(s\right)}$ matrix (Algorithm G in Section S2.4.2 in S1 Appendix). $L^{\left(s\right)}$ is constructed with random eigenvalues drawn from distributions estimated using kernel density estimation over the eigenvalues of ${\hat{L}}^{\text{ME}(1)},\ldots ,{\hat{L}}^{\text{ME}(33)}$. All ${\hat{L}}^{\text{ME}(\ell )}$ are rank 10, and therefore $L^{\left(s\right)}$ is as well. The eigenspace of $L^{\left(s\right)}$ is constructed from the eigenvectors of a random ${\hat{L}}^{\text{ME}(\ell )}$.
2. Generate random Toeplitz matrix, $T^{\left(s\right)}$. $T^{\left(s\right)}$ is defined as equal to a random ${\hat{T}}^{\text{ME}(\ell )}$ without any modification.
3. Define $U^{\left(s\right)}=L^{\left(s\right)}+T^{\left(s\right)}$.

See Section S2.4 in S1 Appendix for further discussion.

We use our polymer model to simulate the corresponding contact map, $H^{\left(s\right)}$, for each $U^{\left(s\right)}$. As described in Section S2.1.6 in S1 Appendix, we simulate chromatin structures via Metropolis Monte Carlo sampling. Each simulation results in 30,000 structures, and $H^{\left(s\right)}$ is calculated from these structures according to Eq (4). At the conclusion of the data generation procedure, we have 5000 pairs $\left(H^{\left(s\right)},U^{\left(s\right)}\right)$.

### Neural network

Generically, graph neural networks (GNNs) require three inputs, (*A*,*X*,*E*). *A* is the adjacency matrix of the graph, *X* is node features, and *E* is the edge features. We use the experimental Hi-C contact map to define all three of these aspects of the graph structure. We include an ablation study demonstrating the importance of the neural network design choices described here in S1 Appendix (Section S2.3.3 and Table A).

We first obtain an experimental contact map, $H^{\text{100kb}}$, binned at 100 kb resolution. While we simulate chromatin at 50 kb resolution, we use 100 kb resolution for the neural network as the GNN both trains faster and performs better when using a coarser resolution (Table A in S1 Appendix). We normalize the contact map by dividing by the mean of its main diagonal. Then we overwrite the main diagonal with 1’s.

We define $A_{ij}=1$ if $H_{ij}^{\text{100kb}}>0$ and $A_{ij}=0$ otherwise. Since we use contact maps at 100 kb resolution, most values $H_{ij}^{\text{100kb}}$ are non-zero, and the graph is nearly complete. If all values are non-zero, the graph will be complete (i.e., *A* will be a matrix of 1’s).

We define node features, *X*, as the top 10 eigenvectors of the 100 kb resolution genomic-distance normalized contact map, ${\tilde{H}}^{\text{100kb}}$. $\tilde{H}$ is calculated by dividing each entry of the contact map by the mean along the corresponding diagonal:


H~ij=Hij mean ⁡ (diagonal ⁡ (H,|i−j|)) (5)

The eigenvectors of this matrix correlate well with biological features such as epigenetic modifications and chromatin compartmentalization [5,6].

We define edge features between nodes *i* and *j* as:


Eij= [log ⁡ Hij100kb,mean ⁡ (diagonal ⁡ (H100kb,|i−j|))]

The first value, log ⁡ Hij100kb, is the log of the corresponding entry of the 100 kb resolution contact map ($E_{ij}$ only needs to be computed when of $A_{ij}=1$, so $H_{ij}^{\text{100kb}}$ is guaranteed to be nonzero.). The second value, $\mathrm{mean}(\mathrm{diagonal}(H^{\text{100kb}},|i$  −  *j* | ) ) , is the mean contact frequency between genomic loci at distance  | *i*  −  *j* | , calculated by taking the mean along the  | *i*  −  *j* | th diagonal of the experimental contact map.

Given these inputs, the GNN outputs $\hat{U}$. We train with mean squared error loss after a log-transformation of *U* and $\hat{U}$, denoted as $U^{†}$ and ${\hat{U}}^{†}$. Specifically, we define $U^{†}:=\text{sign}(U)⊙\ln$  ( | *U* |   +  1 )  where $\text{sign}(U)\in {\left\{-1,1\right\}}^{m\times m}$ contains the element-wise sign of *U* and  ⊙  is the Hadamard (element-wise) product. Computing the loss using the $U^{†}$ representation improves empirical performance (Table A in S1 Appendix).

Details of the training procedure are included in Section S2.3.2 in S1 Appendix.

#### Neural network architecture.

Fig A in S1 Appendix shows a schematic of the architecture. First, the node features are passed through a linear layer to form an initial node embedding (omitted in figure). The node embedding is updated by a series of graph message passing layers. The message passing architecture is a modification of the graph attention network from Brody *et al.* [56] (Section S2.3.1 in S1 Appendix). Between each message passing layer is a multi-layer perception (MLP) with leaky ReLU activations.

The resulting latent node embedding, *Z*, is further processed by two independent modules: an epigenetic module and a genomic-distance module. The epigenetic module computes a bilinear function of the node embedding, $\hat{L}=ZWZ^{T}$, where *W* is a learnable weight matrix. $\hat{L}$ is then upsampled by a factor of two in order to map from 100 kb resolution to 50 kb resolution. The genomic-distance module is an MLP that acts on the flattened node embedding. The output of this MLP is used to construct $\hat{T}$ (at 50 kb resolution). We combine the two modules by adding their results (element-wise) to define $\hat{U}:=\hat{L}+\hat{T}$.

Finally, to account for the sign-invariance of the eigenvector node features, we adopt an idea from the SignNet architecture proposed by Lim *et al.* [57]. The entire GNN architecture is run twice, once with node features defined by positive eigenvectors, *v*, and once by the negated eigenvectors, –*v*, to account for the sign ambiguity of the eigendecomposition. The two resulting predictions are summed to yield a final prediction. We omit this step from Fig A in S1 Appendix for simplicity of presentation.

## Supporting information

S1 Appendix Additional model details and validations. (PDF).

## References

[1] Jerković I, Cavalli G. Understanding 3D genome organization by multidisciplinary methods. Nat Rev Mol Cell Biol 2021;22(8):511–28. doi: 10.1038/s41580-021-00362-w 33953379

[2] Misteli T. The self-organizing genome: principles of genome architecture and function. Cell 2020;183(1):28–45. doi: 10.1016/j.cell.2020.09.014 32976797 PMC7541718

[3] Bintu B, Mateo LJ, Su JH, Sinnott-Armstrong NA, Parker M, Kinrot S, et al. Super-resolution chromatin tracing reveals domains and cooperative interactions in single cells. Science 2018;362(6413):1–8. doi: 10.1126/science.aau1783 PMC6535145 30361340

[4] Su JH, Zheng P, Kinrot SS, Bintu B, Zhuang X. Genome-scale imaging of the 3D organization and transcriptional activity of chromatin. Cell 2020;182(6):1641–59. doi: 10.1016/j.cell.2020.07.032 32822575 PMC7851072

[5] Lieberman-Aiden E, Berkum NLV, Williams L, Imakaev M, Ragoczy T, Telling A, et al. Comprehensive mapping of long-range interactions reveals folding principles of the human genome. Science. 2009;326:289–93. 19815776 10.1126/science.1181369 PMC2858594

[6] Rao SP, Huntley M, Durand N, Stamenova E, Bochkov I, Robinson J, et al. A 3D map of the human genome at kilobase resolution reveals principles of chromatin looping. Cell 2014;159(7):1665–80. doi: 10.1016/j.cell.2014.11.021 25497547 PMC5635824

[7] Rao SSP, Huang SC, Glenn St Hilaire B, Engreitz JM, Perez EM, Kieffer-Kwon KR, et al. Cohesin loss eliminates all loop domains. Cell 2017;171(2):305–20. doi: 10.1016/j.cell.2017.09.026 28985562 PMC5846482

[8] Dixon JR, Selvaraj S, Yue F, Kim A, Li Y, Shen Y, et al. Topological domains in mammalian genomes identified by analysis of chromatin interactions. Nature 2012;485(7398):376–80. doi: 10.1038/nature11082 22495300 PMC3356448

[9] Nora EP, Lajoie BR, Schulz EG, Giorgetti L, Okamoto I, Servant N, et al. Spatial partitioning of the regulatory landscape of the X-inactivation centre. Nature 2012;485(7398):381–5. doi: 10.1038/nature11049 22495304 PMC3555144

[10] Harris HL, Gu H, Olshansky M, Wang A, Farabella I, Eliaz Y, et al. Chromatin alternates between A and B compartments at kilobase scale for subgenic organization. Nat Commun 2023;14(3303):1–17. doi: 10.1038/s41467-023-38429-1 37280210 PMC10244318

[11] Lin X, Qi Y, Latham AP, Zhang B. Multiscale modeling of genome organization with maximum entropy optimization. J Chem Phys 2021;155(1):1–24. doi: 10.1063/5.0044150 PMC8253599 34241389

[12] Oluwadare O, Highsmith M, Cheng J. An overview of methods for reconstructing 3-D chromosome and genome structures from Hi-C data. Biol. Proced. Online 2019;21(1):1–20. doi: 10.1186/s12575-019-0094-0 31049033 PMC6482566

[13] Di Pierro M, Zhang B, Lieberman Aiden E, Wolynes PG, Onuchic JN. Transferable model for chromosome architecture. Proc Natl Acad Sci U S A 2016;113(43):12168–73. doi: 10.1073/pnas.1613607113 27688758 PMC5087044

[14] Le Treut G, Képès F, Orland H. A polymer model for the quantitative reconstruction of chromosome architecture from HiC and GAM data. Biophys J 2018;115(12):2286–94. doi: 10.1016/j.bpj.2018.10.032 30527448 PMC6301988

[15] Qi Y, Zhang B. Predicting three-dimensional genome organization with chromatin states. PLoS Comput Biol 2019;15(6):1–21. doi: 10.1371/journal.pcbi.1007024 PMC6586364 31181064

[16] Zhang B, Wolynes PG. Topology, structures, and energy landscapes of human chromosomes. Proc Natl Acad Sci U S A 2015;112(19):6062–7. doi: 10.1073/pnas.1506257112 25918364 PMC4434716

[17] Moller J, de Pablo JJ. Bottom-up meets top-down: the crossroads of multiscale chromatin modeling. Biophys J 2020;118(9):2057–65. doi: 10.1016/j.bpj.2020.03.014 32320675 PMC7203006

[18] Sun T, Minhas V, Korolev N, Mirzoev A, Lyubartsev AP, Nordenskiöld L. Bottom-up coarse-grained modeling of DNA. Front Mol Biosci. 2021;8:1–17. doi: 10.3389/fmolb.2021.645527 PMC8010198 33816559

[19] Jaynes ET. Information theory and statistical mechanics. Phys Rev. 1957;106(4):620–30.

[20] Schuette G, Ding X, Zhang B. Efficient Hi-C inversion facilitates chromatin folding mechanism discovery and structure prediction. Biophys J 2023;122(17):3425–38. doi: 10.1016/j.bpj.2023.07.017 37496267 PMC10502442

[21] Shin S, Shi G, Thirumalai D. From effective interactions extracted using Hi-C data to chromosome structures in conventional and inverted nuclei. PRX Life. 2023;1(1):1–17.

[22] Shehzad A, Xia F, Member S, Abid S, Peng C, Student Member G, et al. Graph transformers: a survey. arXiv. preprint. 2024; p. 1–23.

[23] Zhou J, Cui G, Hu S, Zhang Z, Yang C, Liu Z, et al. Graph neural networks: a review of methods and applications. AI Open. 2020;1:57–81. doi: 10.1016/j.aiopen.2021.01.001

[24] Vaswani A, Shazeer N, Parmar N, Uszkoreit J, Jones L, Gomez AN, et al. Attention is all you need. arXiv. preprint. 2017; p. 1–15.

[25] Zhang R, Zhou T, Ma J. Multiscale and integrative single-cell Hi-C analysis with Higashi. Nat Biotechnol 2022;40(2):254–61. doi: 10.1038/s41587-021-01034-y 34635838 PMC8843812

[26] Ashoor H, Chen X, Rosikiewicz W, Wang J, Cheng A, Wang P, et al. Graph embedding and unsupervised learning predict genomic sub-compartments from HiC chromatin interaction data. Nat Commun 2020;11(1):1–11. doi: 10.1038/s41467-020-14974-x 32127534 PMC7054322

[27] Brown TB, Mann B, Ryder N, Subbiah M, Kaplan J, Dhariwal P, et al. Language models are few-shot learners. Adv Neural Inf Process Syst. 2020;33:1877–901. doi: 10.48550/arXiv.2005.14165

[28] OpenAI. GPT-4 technical report. arXiv. preprint. 2023; p. 2303–08774.

[29] Akdel M, Pires DEV, Pardo EP, Jänes J, Zalevsky AO, Mészáros B, et al. A structural biology community assessment of AlphaFold2 applications. Nat Struct Mol Biol 2022;29(11):1056–67. doi: 10.1038/s41594-022-00849-w 36344848 PMC9663297

[30] Jumper J, Evans R, Pritzel A, Green T, Figurnov M, Ronneberger O, et al. Highly accurate protein structure prediction with AlphaFold. Nature 2021;596(7873):583–589. doi: 10.1038/s41586-021-03819-2 34265844 PMC8371605

[31] Kryshtafovych A, Schwede T, Topf M, Fidelis K, Moult J. Critical assessment of methods of protein structure prediction (CASP)—Round XIV. Proteins 2021;89(12):1607–17. doi: 10.1002/prot.26237 34533838 PMC8726744

[32] Yang T, Zhang F, Yardımci GG, Song F, Hardison RC, Noble WS, et al. HiCRep: assessing the reproducibility of Hi-C data using a stratum-adjusted correlation coefficient. Genome Res 2017;27(11):1939–49. doi: 10.1101/gr.220640.117 28855260 PMC5668950

[33] Yan KK, Yardlmcl GG, Yan C, Noble WS, Gerstein M. HiC-spector: a matrix library for spectral and reproducibility analysis of Hi-C contact maps. Bioinformatics 2017;33(14):2199–201. doi: 10.1093/bioinformatics/btx152 28369339 PMC5870694

[34] Kadam S, Kumari K, Manivannan V, Dutta S, Mitra MK, Padinhateeri R. Predicting scale-dependent chromatin polymer properties from systematic coarse-graining. Nat Commun 2023;14(1):1–14. doi: 10.1038/s41467-023-39907-2 37433821 PMC10336007

[35] Nagano T, Lubling Y, Stevens TJ, Schoenfelder S, Yaffe E, Dean W, et al. Single-cell Hi-C reveals cell-to-cell variability in chromosome structure. Nature 2013;502(7469):59–64. doi: 10.1038/nature12593 24067610 PMC3869051

[36] Galitsyna AA, Gelfand MS. Single-cell Hi-C data analysis: safety in numbers. Brief Bioinf 2021;22(6):1–13. doi: 10.1093/bib/bbab316 PMC8575028 34406348

[37] Zhou J, Ma J, Chen Y, Cheng C, Bao B, Peng J, et al. Robust single-cell Hi-C clustering by convolution- and random-walk–based imputation. Proc Natl Acad Sci U S A 2019;116(28):14011–18. doi: 10.1073/pnas.1901423116 31235599 PMC6628819

[38] Gkeka P, Stoltz G, Barati Farimani A, Belkacemi Z, Ceriotti M, Chodera JD, et al. Machine learning force fields and coarse-grained variables in molecular dynamics: application to materials and biological systems. J. Chem. Theory Comput 2020;16(8):4757–75. doi: 10.1021/acs.jctc.0c00355 32559068 PMC8312194

[39] Noid WG. Perspective: coarse-grained models for biomolecular systems. J Chem Phys 2013;139(9):1–25. doi: 10.1063/1.4818908 24028092

[40] Han B, Yu K. Refining potential energy surface through dynamical properties via differentiable molecular simulation. Nat Commun 2025;16(1):816. doi: 10.1038/s41467-025-56061-z 39827185 PMC11742923

[41] Navarro C, Majewski M, De Fabritiis G. Top-down machine learning of coarse-grained protein force fields. J Chem Theory Comput 2023;19(21):7518–26. doi: 10.1021/acs.jctc.3c00638 37874270 PMC10777392

[42] Thaler S, Zavadlav J. Learning neural network potentials from experimental data via differentiable trajectory reweighting. Nat Commun 2021;12(1):6884. doi: 10.1038/s41467-021-27241-4 34824254 PMC8617111

[43] Wang W, Wu Z, Dietschreit JCB, Gómez-Bombarelli R. Learning pair potentials using differentiable simulations. J Chem Phys. 2023;158(4). doi: 10.1063/5.0126475 36725529

[44] Durand NC, Robinson JT, Shamim MS, Machol I, Mesirov JP, Lander ES, et al. Juicebox provides a visualization system for Hi-C contact maps with unlimited zoom. Cell Syst 2016;3(1):99–101. doi: 10.1016/j.cels.2015.07.012 27467250 PMC5596920

[45] Hitz BC, Lee JW, Jolanki O, Kagda MS, Graham K, Sud P, et al. The ENCODE uniform analysis pipelines. bioRxiv. preprint. 2023. doi: 10.1101/2023.04.04.535623

[46] Kagda MS, Lam B, Litton C, Small C, Sloan CA, Spragins E, et al. Data navigation on the ENCODE portal—introduction. arXiv. preprint. 2023; p. 1–39. 10.1038/s41467-025-64343-9 PMC12575607 41168159

[47] Luo Y, Hitz BC, Gabdank I, Hilton JA, Kagda MS, Lam B, et al. New developments on the Encyclopedia of DNA Elements (ENCODE) data portal. Nucleic Acids Res. 2020;48(D1):D882–9. doi: 10.1093/nar/gkz1062 31713622 PMC7061942

[48] The ENCODE Project Consortium. An integrated encyclopedia of DNA elements in the human genome. Nature 2012;489(7414):57–74. doi: 10.1038/nature11247 22955616 PMC3439153

[49] Imakaev M, Fudenberg G, McCord RP, Naumova N, Goloborodko A, Lajoie BR, et al. Iterative correction of Hi-C data reveals hallmarks of chromosome organization. Nat Methods 2012;9(10):999–1003. doi: 10.1038/nmeth.2148 22941365 PMC3816492

[50] Knight PA, Ruiz D. A fast algorithm for matrix balancing. IMA J Numer Anal 2013;33(3):1029–47. doi: 10.1093/imanum/drs019

[51] Detcheverry FA, Pike DQ, Nagpal U, Nealey PF, De Pablo JJ. Theoretically informed coarse grain simulations of block copolymer melts: method and applications. Soft Matter 2009;5(24):4858–65. doi: 10.1039/b911646j

[52] Detcheverry FA, Kang H, Daoulas KC, Müller M, Nealey PF, De Pablo JJ. Monte Carlo simulations of a coarse grain model for block copolymers and nanocomposites. Macromolecules 2008;41(13):4989–5001. doi: 10.1021/ma702514v

[53] Pike DQ, Detcheverry FA, Müller M, De Pablo JJ. Theoretically informed coarse grain simulations of polymeric systems. J Chem Phys 2009;131(8):1–10. doi: 10.1063/1.3187936 19725633

[54] MacPherson Q, Beltran B, Spakowitz AJ. Bottom–up modeling of chromatin segregation due to epigenetic modifications. Proc Natl Acad Sci U S A 2018;115(50):12739–44. doi: 10.1073/pnas.1812268115 30478042 PMC6294944

[55] Shi G, Thirumalai D. A maximum-entropy model to predict 3D structural ensembles of chromatin from pairwise distances with applications to interphase chromosomes and structural variants. Nat Commun 2023;14(1):1150. doi: 10.1038/s41467-023-36412-4 36854665 PMC9974990

[56] Brody S, Alon U, Yahav E. How attentive are graph attention networks? arXiv. preprint. 2022.

[57] Lim D, Robinson J, Zhao L, Smidt T, Sra S, Maron H, et al. Sign and basis invariant networks for spectral graph representation learning. arXiv. preprint. 2022; pp. 1–42.

