# Supplementary material for: Chromatin structures from integrated AI and polymer physics model
Source: PLoS Comput Biol. 2025 Apr 9;21(4):e1012912. doi: 10.1371/journal.pcbi.1012912 (PMC12005555; doi:10.1371/journal.pcbi.1012912)
Supplement: S1 Appendix — (PDF). [file pcbi.1012912.s001.pdf]

# Supporting Information for Chromatin structures from integrated AI and polymer physics model

Eric R Schultz[1], Soren Kyhl[1], Rebecca Willett[2], Juan J de Pablo[1,3,4*]

**1** Pritzker School of Molecular Engineering, The University of Chicago, Chicago, Illinois, United States of America
**2** Department of Statistics and Computer Science, The University of Chicago, Chicago, Illinois, United States of America
**3** Tandon School of Engineering, New York University, Brooklyn, New York, United States of America
**4** Materials Science Division, Argonne National Laboratory, Lemont, Illinois, United States of America

* j.de.pablo@nyu.edu

## S1 Pseudocode

Algorithm A defines our simulation procedure. See Section S2.1.6 for a description of the Monte Carlo sampling procedure. We use this simulation procedure for both the maximum entropy approach (Algorithm D) and the GNN approach (Algorithm E). Algorithm B defines how we generate simulated contact maps. Algorithm D defines the maxim entropy optimization approach for estimating simulation parameters. Line 7 calls our Newton's method procedure, which we don't define for simplicity. See Section S2.2.5 for details of the maximum entropy optimization. Algorithm E defines the GNN approach for estimating simulation parameters. Line 1 calls our graph neural network, which we don't define for simplicity.

**Algorithm A** $H^{\text{sim}} \leftarrow$ SimulationEngine

**Input** $\mathcal{U} \in \mathbb{R}^{m \times m}$, net energy matrix
$n$, number of structures to simulate

**for** $l = 1, \ldots, n$ **do**
  $\vec{r}^{(l)} \in \mathbb{R}^{m \times 3} \sim p(\vec{r} : \mathcal{U})$ ▷ sample from probability distribution via Monte Carlo sampling
**end for**
$H^{\text{sim}} \leftarrow \text{ContactMap}(\vec{r}^{(1)}, \ldots, \vec{r}^{(n)})$
**return** $H^{\text{sim}}$

**Algorithm B** $H^{\text{sim}} \leftarrow$ ContactMap

**Input** $\vec{r}^{(1)}, \ldots, \vec{r}^{(n)} \in \mathbb{R}^{m \times 3}$, simulated structures
**Parameter** $\Delta$, grid size

$H^{\text{sim}} \leftarrow 0^{m \times m}$ ▷ initialize contact map to zeros
**for** $l = 1, \ldots, n$ **do**
  **for** $i = 1, \ldots, m$ **do**
    **for** $j = i, \ldots, m$ **do**
      $c_i \leftarrow \text{GetGridCell}(\vec{r}_i^{(\ell)}, \Delta)$
      $c_j \leftarrow \text{GetGridCell}(\vec{r}_j^{(\ell)}, \Delta)$
      $H_{ij}^{\text{sim}} \leftarrow H_{ij}^{\text{sim}} + \delta_{c_i, c_j}$ ▷ increment contact map if particles are in same grid cell
      ▷ where $\delta$ is the Kronecker delta
    **end for**
  **end for**
**end for**
**return** $H^{\text{sim}} \in {\mathbb{Z}^+}^{m \times m}$, simulated contact map ▷ where $\mathbb{Z}^+$ is the set of positive integers

**Algorithm C** $c \leftarrow$ GetGridCell

**Input** $\vec{r}_i \in \mathbb{R}^3$, position of particle $i$
$\Delta$, grid size

$c_i \leftarrow (\lfloor \vec{r}_{i1}/\Delta \rfloor, \lfloor \vec{r}_{i2}/\Delta \rfloor, \lfloor \vec{r}_{i3}/\Delta \rfloor)$
**return** $c_i$, 3-tuple indicating grid cell of particle $i$

**Algorithm D** $H^{\mathrm{sim}} \leftarrow$ MaximumEntropyOptimizationApproach

**Input** $H^{\mathrm{exp}} \in \mathbb{Z}^{+m \times m}$, experimental contact map
$\epsilon$, convergence criterion
$n$, number of structures to simulate per iteration

$i \leftarrow 1$ ▷ initialize iteration to 0
$\hat{\mathcal{U}}^{\mathrm{ME}} \leftarrow 0^{m \times m}$ ▷ initialize net energy matrix to zeros
$\ell_0 \leftarrow \mathrm{inf}$ ▷ initialize loss to infinity
$\Delta\ell \leftarrow \mathrm{inf}$ ▷ initialize delta loss to infinity
**while** $\Delta\ell > \epsilon$ **do**
  $H^{\mathrm{sim}} \leftarrow$ SimulationEngine$(\hat{\mathcal{U}}^{\mathrm{ME}}, n)$
  $\hat{\mathcal{U}}^{\mathrm{ME}}, \ell_i \leftarrow$ NewtonsMethod$(\hat{\mathcal{U}}^{\mathrm{ME}}, H^{\mathrm{exp}}, H^{\mathrm{sim}})$ ▷ update $\hat{\mathcal{U}}^{\mathrm{ME}}, \ell$ with Newton's method
  $\Delta\ell \leftarrow \ell_i - \ell_{i-1}$
  $i \leftarrow i + 1$
**end while**
$H^{\mathrm{sim}} \leftarrow$ SimulationEngine, $(\hat{\mathcal{U}}^{\mathrm{ME}}, n)$ ▷ run simulation with final net energy matrix
**return** $H^{\mathrm{sim}}$

**Algorithm E** $H^{\mathrm{sim}} \leftarrow$ GNNApproach

**Input** $H^{\mathrm{exp}} \in \mathbb{Z}^{+m \times m}$, experimental contact map
$n$, number of structures to simulate

$\hat{\mathcal{U}}^{\mathrm{GNN}} \leftarrow$ GNN$(H^{\mathrm{exp}})$ ▷ use graph neural network to predict net energy matrix
$H^{\mathrm{sim}} \leftarrow$ SimulationEngine$(\hat{\mathcal{U}}, n)$ ▷ run simulation with predicted net energy matrix
**return** $H^{\mathrm{sim}}$

# S2 Supplementary Methods

## S2.1 Polymer Model Details

Similar to other top-down approaches for chromatin modeling [7, 24, 27, 35], we employ a generic coarse-grain heteropolymer model as the basis for our polymer model. Then, we build in additional complexity using experimental data as constraints in a top-down fashion. We treat 50 kb of DNA as one simulation particle. The energy functional of our polymer model can be decomposed as a sum of bonded, $U^{\mathrm{b}}(\vec{r})$, and nonbonded, $U^{\mathrm{nb}}(\vec{r})$, contributions:

$$\frac{U(\vec{r})}{k_B T} = \frac{U^{\mathrm{b}}(\vec{r})}{k_B T} + \frac{U^{\mathrm{nb}}(\vec{r})}{k_B T} \tag{1}$$

where $\vec{r} \in \mathbb{R}^{m\times 3}$ contains the positions of all $m$ particles in the polymer, $k_B$ is the Boltzmann constant, and $T$ is temperature.

In a slight abuse of notation, we omit $\frac{1}{k_B T}$ from the remainder of the equations in this manuscript for simplicity.

The bonded interaction controls the interaction between particles $i$ and $i+1$. We use a Gaussian chain bonded interaction defined as follows:

$$U^{\mathrm{b}}(\vec{r}) = \sum_{i\in\{1,\ldots,m\}} \frac{3}{2b^2}(\vec{r}_i - \vec{r}_{i+1})^2 \tag{2}$$

where $b$ is the desired mean bond length and $\vec{r}_i$ is the position of the $i$th particle.

The non-bonded interaction describes the interaction between pairs of particles and can be further decomposed into an epigenetic, $U^{\mathrm{epi}}(\vec{r})$, and genomic distance, $U^{\mathrm{dist}}(\vec{r})$, contribution.

$$U^{\mathrm{nb}}(\vec{r}) = U^{\mathrm{epi}}(\vec{r}) + U^{\mathrm{dist}}(\vec{r}) \tag{3}$$

### S2.1.1 Epigenetic Contribution

The epigenetic contribution reproduces phase separation between particles with different chromatin states. For now, consider each particle to be labeled with a mutually exclusive chromatin state label. In particular, we use the Theoretically Informed Coarse Grain (TICG) potential [5, 6, 23]. The TICG potential is a mean-field technique originally developed within the de Pablo group to study polymer phase separation. Notably, the TICG potential has previously been used to model chromatin structure, although in a bottom-up fashion [19].

The TICG potential calculates nonbonded interactions via a field-theoretic Hamiltonian based on the local density of the polymer calculated upon a grid-based discretization of cartesian space.

$$U^{\mathrm{epi}}(\vec{r}) = \sum_{c\in\mathrm{grid}} \sum_{\substack{I\geq J \\ I,J\in\mathrm{labels}}} \chi_{IJ} \frac{\Delta^3}{V_p} \phi_{I,c}\phi_{J,c} \tag{4}$$

where $\chi_{IJ}$ is the Flory-Huggins interaction energy between particles with label $I$ and with label $J$, $\phi_{I,c}$ is the volume fraction of particles with label $I$ in grid cell $c$ for a given configuration $\vec{r}$, $\Delta$ is the grid size, $V_p$ is the volume of an individual particle.

The advantage of the TICG functional is that the structural ensemble can be efficiently sampled via Monte Carlo sampling (detailed in Section S2.1.6) in $\mathcal{O}(m)$ time where $m$ is the number of particles. As a result, our simulation is sufficiently efficient to enable the simulation of entire chromosomes at nucleosome resolution (200 bp) [19]. However, in this work, we use a resolution of 50 kb as a proof of concept of our approach.

#### S2.1.2 Genomic Distance Contribution

The genomic distance contribution reproduces the contact probability scaling seen in experimental Hi-C data. This term is inspired by and analogous to the "ideal chromosome" term in the MiChrom introduced by Di Pierro *et al.* [7]. By design, this term is invariant to translation along the genome and is independent of the epigenetic contribution.

$$U^{\text{dist}}(\vec{r}) = \sum_{c\in\text{grid}} \sum_{d=1}^{m} \chi_d^{\text{dist}} \frac{\Delta^3}{V_p} \sqrt{2\phi_{d,c}} \tag{5}$$

where $d = |i-j|$ is the distance along the polymer chain between particles $i$ and $j$, $\chi_d^{\text{dist}} > 0$ is the interaction energy between particles with genomic separation $d$, $\phi_{d,c}$ is the volume fraction of *bonds* between particles of genomic distance $d$ in grid cell $c$ for a given configuration $\vec{r}$.

#### S2.1.3 Net Interaction Representation

To make the machine learning task more tractable we re-write $U^{\text{nb}}$. A brief outline of the derivation is shown here, and a full derivation is included below in Section S2.1.4.

The epigenetic contribution, $U^{\text{epi}}$, can be rewritten as:

$$U^{\text{epi}}(\vec{r}) = \sum_{i,j\in\{1,\dots,m\}} I(\vec{r}_i, \vec{r}_j) \frac{\Psi_i^T \chi \Psi_j V_p}{\Delta^3} \tag{6}$$

where $I(\vec{r}_i, \vec{r}_j) \in \{0, 1\}$ indicates if particle $i$ and $j$ are in the same grid cell, $\Psi_i = \{0, 1\}^k$ is a binary vector containing the labels of particle $i$, and $\chi$ is an upper triangular matrix containing every $\chi_{I,J}$

In contrast to Eq. (4), which was a double sum over all grid cells and all labels, Eq. (6) is a sum over all pairs of beads. In Eq. (4), the particles have discrete labels, and therefore $\Psi_i$ is a binary vector. However, $\Psi_i$ can be real-valued in general. For convenience, we define $L_{ij} = (\Psi_i^T \chi \Psi_j + \Psi_j^T \chi \Psi_i)/2$. $L_{ij}$ contains the net epigenetic interaction energy between particles $i$ and $j$ and is symmetric by construction. $L$ can be factorized to recover $\chi$ if the particle labels, $\Psi$, are known.

$$U^{\text{epi}}(\vec{r}) = \sum_{i,j\in\{1,\dots,m\}} I(\vec{r}_i, \vec{r}_j) \frac{L_{ij} V_p}{\Delta^3} \tag{7}$$

where $L_{ij}$ is the net epigenetic interaction between particles $i$ and $j$.

By a similar approach, the genomic distance contribution, $U^{\text{dist}}$, can be rewritten as:

$$U^{\text{dist}}(\vec{r}) = \sum_{i,j\in\{1,\dots,m\}} I(\vec{r}_i, \vec{r}_j) \frac{T_{ij} V_p}{\Delta^3} \tag{8}$$

where $T_{ij} := \chi_{d=|i-j|}^{\text{dist}}$ is the genomic distance interaction between particles $i$ and $j$.

By construction, $T$ has a main diagonal of zeros, and all descending diagonals are constant-valued. Such a matrix is referred to as a Toeplitz matrix.

We can combine the genomic distance (8) and epigenetic contributions (7) into a single non-bonded term:

$$U^{\text{nb}}(\vec{r}) = \sum_{i,j\in\{1,\dots,m\}} I(\vec{r}_i, \vec{r}_j) \frac{(L_{ij} + T_{ij}) V_p}{\Delta^3} \tag{9}$$

#### S2.1.4 Derivation

Consider Eq. (4):

$$U^{\mathrm{epi}}(\vec{r}) = \sum_{c \in \mathrm{grid}} \sum_{\substack{I \geq J \\ I,J \in \mathrm{labels}}} \chi_{IJ} \frac{\Delta^3}{V_p} \phi_{I,c} \phi_{J,c}$$

where $\chi_{IJ}$ is the Flory-Huggins interaction energy between particles with label $I$ and with label $J$, $\phi_{I,c}$ is the volume fraction of particles with label $I$ in grid cell $c$ for a given configuration $\vec{r}$, $\Delta$ is the grid size, and $V_p$ is the volume of an individual particle.

By definition:

$$\phi_{I,c} = \sum_{i=1}^{m} \mathbb{1}_{i \in c} \frac{\Psi_{i,I} V_p}{\Delta^3}$$

where $\mathbb{1}_{i \in c} = 1$ if particle $i$ is in grid cell $c$ (0 otherwise) and $\Psi_{i,I} = 1$ if particle $i$ has label $I$ (0 otherwise).

By substituting $\phi_{I,c}$:

$$U^{\mathrm{epi}}(\vec{r}) = \sum_{c \in \mathrm{grid}} \sum_{\substack{I \geq J \\ I,J \in \mathrm{Labels}}} \chi_{IJ} \frac{\Delta^3}{V_p} \sum_{i=1}^{m} \mathbb{1}_{i \in c} \frac{\Psi_{i,I} V_p}{\Delta^3} \sum_{j=1}^{m} \mathbb{1}_{j \in c} \frac{\Psi_{j,J} V_p}{\Delta^3}$$

Which can be rewritten as:

$$U^{\mathrm{epi}}(\vec{r}) = \sum_{c \in \mathrm{grid}} \sum_{i,j \in \{1,\ldots,m\}} \mathbb{1}_{i \in c} \mathbb{1}_{j \in c} \frac{\Psi_i^T \chi \Psi_j V_p}{\Delta^3}$$

where $\sum_{i,j \in \{1,\ldots,m\}}$ is a sum over all pairs of particles $(i,j)$ and $\chi$ is an upper triangular matrix containing every $\chi_{I,J}$.

Which can be rewritten as Eq. (6):

$$U^{\mathrm{epi}}(\vec{r}) = \sum_{i,j \in \{1,\ldots,m\}} I(\vec{r}_i, \vec{r}_j) \frac{\Psi_i^T \chi \Psi_j V_p}{\Delta^3}$$

where $I(\vec{r}_i, \vec{r}_j)$ indicates if particle $i$ and $j$ are in the same grid cell.

Define $L_{ij} := (\Psi_i^T \chi \Psi_j + \Psi_j^T \chi \Psi_i)/2$. Then, $L_{ij}$ contains the net epigenetic interaction energy between particles $i$ and $j$ and is symmetric by construction (Eq. (7)):

$$U^{\mathrm{epi}}(\vec{r}) = \sum_{i,j \in \{1,\ldots,m\}} I(\vec{r}_i, \vec{r}_j) \frac{L_{ij} V_p}{\Delta^3}$$

where $L_{ij}$ is the net epigenetic interaction between particles $i$ and $j$.

Now consider Eq. (5):

$$U^{\mathrm{dist}}(\vec{r}) = \sum_{c \in \mathrm{grid}} \sum_{d=1}^{m} \chi_d^{\mathrm{dist}} \frac{\Delta^3}{V_p} \phi_{d,c}$$

where $d = |i - j|$ is the distance along the polymer chain between particles $i$ and $j$, $\chi_d^{\mathrm{dist}} > 0$ is the interaction energy between particles with genomic separation $d$, and $\phi_{d,c}$ is the volume fraction of *bonds* between particles of genomic distance $d$ in grid cell $c$.

By definition:

$$\phi_{d,c} = \sum_{i=1}^{m}\sum_{j=1}^{m} \mathbb{1}_{i\in c}\mathbb{1}_{j\in c}\mathbb{1}_{|i-j|=d}\frac{(V_p)^2}{\Delta^6}$$

By substitution, the following is true:

$$U^{\text{dist}}(\vec{r}) = \sum_{\substack{i\neq j\\ i,j\in\{1,\ldots,m\}}} I(\vec{r}_i,\vec{r}_j)\frac{\chi_d^{\text{dist}}V_p}{\Delta^3}$$

We can rewrite this equation by defining $T_{ij} = \chi^{\text{dist}}_{d=|i-j|}$ and $T_{ii} = 0$ (Eq. (8)):

$$U^{\text{dist}}(\vec{r}) = \sum_{i,j\in\{1,\ldots,m\}} I(\vec{r}_i,\vec{r}_j)\frac{T_{ij}V_p}{\Delta^3}$$

Finally, we can clearly see that the genomic-distance dependent (Eq. (8)) and epigenetic effects (Eq. (7)) can be combined as follows into a single non-bonded term (Eq. (9)):

$$U^{\text{nb}}(\vec{r}) = \sum_{i,j\in\{1,\ldots,m\}} I(i,j)\frac{(L_{ij}+T_{ij})V_p}{\Delta^3}$$

Alternatively,

$$U^{\text{nb}}(\vec{r}) = \sum_{\substack{i\geq j\\ i,j\in\{1,\ldots,m\}}} I(i,j)\frac{(L^*_{ij}+2T_{ij})V_p}{\Delta^3} \tag{10}$$

where $L^* = 2L - I_m \odot (L)$, where $\odot$ is the Hadamard product and $I_m$ is the size $m$ identity matrix.

Define $\mathcal{U} = L + T$ and $\mathcal{U}^* = L^* + 2T$. Eq. (10) is more efficient than Eq. (9) since it only considers pairs of particles where $i \geq j$. For this reason, we use Eq. (10) during the Monte Carlo simulation.

#### S2.1.5 User-Defined Parameters

All simulations described in the main text use a particle volume, $V_p$, of 130,000 nm$^3$, a bond length, $b$, of 200 nm, a simulation volume, $V$, of 8 $\mu$m$^3$, and the simulation boundary is a prolate spheroid with an aspect ratio of 1.5. The GNN is trained on a simulated dataset using these bonded parameters, and therefore, it may not extrapolate to other choices of bonded parameters. However, the GNN does not depend in any way on the choice of bonded parameter; we could train a GNN on simulated data using any reasonable choice of bonded parameters.

The particle volume, $V_p$, is defined based on the resolution of the simulated chromatin fiber. A coarse-grained particle at nucleosome resolution (200 bp per particle) is assumed to have a particle volume of 520 nm$^3$ as in the polymer model of MacPherson *et al.* [19]. Since we use 50 kb resolution for all simulations, we rescale the particle volume using a Gaussian renormalization [26], resulting in a particle volume of 130,000 nm$^3$.

We choose the bond length, $b$, simulation volume, $V$, and aspect ratio based on the experimental distance scaling (Fig. D) from Su et al. [28]. Using ($b$ = 200 nm, $V$ = 8 $\mu$m$^3$, aspect ratio = 1.5) yields the best reproduction of the distance scaling as a function of genomic distance. Interestingly, we find that a prolate boundary better reproduces experimental distance scaling than a spherical boundary (Fig. DC). This result is consistent with experimental evidence that chromosome structures are aspherical [14].

The grid size, $\Delta$, is chosen such that a simulation with only bonded interactions matches the average experimental contact frequency between adjacent particles ($i$ and $i \pm 1$). Since all experimental contact maps

are normalized such that the average contact frequency between adjacent particles is 0.1, we use the same grid size for all simulations ($\Delta = 151.6$ nm).

Within the maximum entropy approach, the particle labels, $\Psi$ in Eq. (6), are user-defined parameters. Prior works choose the labels to represent 'chromatin states', which are binary and mutually exclusive [7, 24]. The default TICG epigenetic term (Eq. (4)) assumes that particles have binary labels but does not require that these labels be mutually exclusive. Further, rewriting the epigenetic term as in Eq. (6) enables us to use real-valued labels. We use the first $k = 10$ principal components of the genomic-distance normalized contact map, $\tilde{H}$, as the particle labels. We choose $k = 10$ because the simulation performance begins to plateau for $k > 10$ when using the maximum entropy approach (Fig. G). Note that the GNN does not require $\Psi$ as an input, and therefore does not depend on $k$. However, for ease of comparison to the maximum entropy approach, we only train the GNN on simulated data where the epigenetic interaction matrix, $L$, is rank $k = 10$.

### S2.1.6 Monte Carlo Simulation

We sample the equilibrium ensemble of our polymer model with Metropolis Monte Carlo sampling. Simulations are run for 300,000 Monte Carlo cycles. We employ four Monte Carlo moves: 1) Displacement, 2) Translation, 3) Crankshaft, and 4) Pivot. A Monte Carlo cycle consists of $m$ Displacement, 500 Translation, 500 Crankshaft, and 50 Pivot moves, where $m$ is the number of particles. We record the current configuration every 10 cycles, resulting in 30,000 structures per simulation.

Displacement translates a single random particle by 5 nm in a random direction.

Translation displaces a random polymer segment by 30% of the bond length in a random direction. The random polymer segment is defined by first choosing a random particle, then by choosing a second random particle from a two-sided exponential distribution with scale parameter $\frac{m}{100}$.

Crankshaft rotates a random polymer segment about the axis defined by its ends. The random polymer segment is chosen as in Translation, except the polymer segment cannot contain the first or last bead. The polymer segment is then rotated by a random rotation in the interval $[-\frac{\pi}{12}, \frac{\pi}{12}]$ radians.

Pivot rotates one end of the polymer about a random axis. One of the two polymer ends is chosen randomly, and a random particle is chosen. The resulting polymer end segment is rotated by a random rotation in the interval $[-\frac{\pi}{12}, \frac{\pi}{12}]$ radians around a randomly chosen axis.

In addition to these moves, we reposition the simulation grid during each Monte Carlo cycle. This prevents the discrete grid implementation from causing simulation artifacts at grid boundaries.

If any Monte Carlo move causes any specific grid cell to have a chromatin volume fraction greater than 0.5, we reject that move.

## S2.2 Maximum Entropy Optimization

### S2.2.1 Overview

The values of $\chi_{IJ}$ in Eq. (4) and $\chi_d^{\text{dist}}$ in Eq. (5) are not known *a priori* and must be inferred from experimental data. The maximum entropy principle is a common optimization framework for inferring unknown parameters from experimental data [12]. This approach has been applied previously to chromatin simulations (reviewed in [17]) and we use it as a baseline for comparison to our neural network-based approach.

In the maximum entropy approach, a model, $U^{\text{ME}}$, is learned as a correction to a more naive model. In our polymer model, we define $U^{\text{ME}}(\vec{r}; \chi_{IJ}, \chi_d^{\text{dist}}) := U^{\text{b}}(\vec{r}) + U^{\text{nb}}(\vec{r}; \chi_{IJ}, \chi_d^{\text{dist}})$. The homopolymer bonded term, $U^{\text{b}}$, is the naive model, and the nonbonded term, $U^{\text{nb}}$, is the maximum entropy correction term.

We seek to estimate $\chi_{IJ}$ and $\chi_d^{\text{dist}}$ such that user-defined experimental observables, $\nu_i^{\text{exp}}$, match their simulated counterparts:

$$\nu_i^{\text{sim}} := \langle \nu_i \rangle_{\vec{r}} := \frac{\int \nu_i(\vec{r}) e^{-\beta U_{\text{ME}}(\vec{r}; \chi_{IJ}, \chi_d^{\text{dist}})} d\vec{r}}{\int e^{-\beta U_{\text{ME}}(\vec{r}; \chi_{IJ}, \chi_d^{\text{dist}})} d\vec{r}}$$

We define our experimental observables based on the experimental contact map (Section S2.2.3). We will optimize $\chi_{IJ}$ and $\chi_d^{\text{dist}}$ subject to the constraint that $\nu_i^{\text{sim}} = \nu_i^{\text{exp}}$ (Section S2.2.5). Note that the constraints are not guaranteed to hold with equality. Specifically, we seek to maximize the information entropy subject to our constraints:

$$s = -\int \pi^{\text{ME}}(\vec{r}) ln\big(\pi^{\text{ME}}(\vec{r})\big) d\vec{r}$$

where $\pi^{\text{ME}}$ is the maximum entropy probability density.

Our description of the maximum entropy approach is adapted from the supplement of di Pierro *et al.* [7].

#### S2.2.2 Notation

$m$ is the number of particles in the simulation

$H^{\text{exp}} \in \mathbb{Z}^{+m \times m}$ is an experimental Hi-C contact map ($\mathbb{Z}^+$ is the set of positive integers)

$U^{\text{b}}(\vec{r})$ is a simple homopolymer (bonded) energy functional - we use a Gaussian chain potential

$\vec{r} \in \mathbb{R}^{m \times 3}$ contains the coordinates of all m particles in 3D space

$H^{\text{inst}}(\vec{r}) \in \mathbb{Z}^{+m \times m}$ is a *instance* simulated contact map (i.e., corresponding to a single configuration $\vec{r}$)

$H^{\text{sim}} := \langle H^{\text{inst}} \rangle_{\vec{r}} \in \mathbb{Z}^{+m \times m}$ is the *ensemble* simulated contact map (i.e., the ensemble average over $\vec{r}$)

$\Psi \in \mathbb{R}^{m \times k}$ are the particle labels

$\psi_{:,I} \in \mathbb{R}^m$ is a column in $\Psi$

#### S2.2.3 ME Problem Formulation

Given $H^{\text{exp}}$, $U^{\text{b}}(\vec{r})$, and $\Psi$ the maximum entropy procedure seeks a modification of $U^{\text{b}}(\vec{r})$ such that $H^{\text{sim}}$ approximates $H^{\text{exp}}$.

More explicitly, the maximum entropy (ME) procedure seeks to find a probability distribution ($\pi^{\text{ME}}$) such that the following constraints hold:

$$0 = c_0 := \int \pi^{\text{ME}}(\vec{r}) d\vec{r} - 1$$

$$0 = c_1 := \int U^{\text{b}}(\vec{r}) \pi^{\text{ME}}(\vec{r}) d\vec{r} - \frac{3}{2} m k_B T$$

$$0 = c_{IJ}^{\text{epi}} := \int \psi_{:,I}^T H^{\text{inst}}(\vec{r}) \psi_{:,J} \pi^{\text{ME}}(\vec{r}) d\vec{r} - \psi_{:,I}^T H^{\text{exp}} \psi_{:,J} \text{ for } I, J \in \{1, \ldots, k\} \mid I \leq J$$

$$0 = c_d^{\text{dist}} := \int \sum_{i=d+1}^{N-d} H_{i,i+N}^{\text{inst}}(\vec{r}) \pi^{\text{ME}}(\vec{r}) d\vec{r} - \sum_{i=d+1}^{N-d} H_{i,i+m}^{\text{exp}} \text{ for } d = 1, \ldots, m-1$$

where $c_0$ constrains the distribution to be normalized, $c_1$ constrains the average potential energy to be equal to the thermal energy (where $k_B$ is the Boltzmann constant and T is temperature), $c_{IJ}^{\text{epi}}$ constrains the average contact frequency between beads of type $I$ and type $J$ to be the same in the experiment and the simulation, and $c_d^{\text{dist}}$ constrains the average contact frequency along each diagonal to be the same in the experiment and the simulation (in practice, we typically coarse-grain $c_d^{\text{dist}}$ from $d$ constraints to 96 constraints).

#### S2.2.4 Derivation of ME Probability Distribution

To optimize the parameters of $\pi^{\mathrm{ME}}$, we seek to maximize the information entropy subject to our constraints:

$$s = -\int \pi^{\mathrm{ME}}(\vec{r}) \ln\big(\pi^{\mathrm{ME}}(\vec{r})\big) d\vec{r}$$

Using Lagrange multipliers we obtain the following extremum condition:

$$\frac{\partial s}{\partial \pi^{\mathrm{ME}}} - \lambda_0 \frac{\partial c_0}{\partial \pi^{\mathrm{ME}}} - \lambda_1 \frac{\partial c_1}{\partial \pi^{\mathrm{ME}}} - \lambda_{IJ}^{\mathrm{epi}} \sum_{I,J} \frac{\partial c_{IJ}^{\mathrm{epi}}}{\partial \pi^{\mathrm{ME}}} - \lambda_d^{\mathrm{dist}} \sum_{d} \frac{\partial c_d^{\mathrm{dist}}}{\partial \pi^{\mathrm{ME}}} = 0$$

where each Lagrange multiplier, $\lambda$, corresponds to one of the constraints defined above.
Omitting the intermediary steps of the derivation, and renaming the following Lagrange multipliers:

$$\beta := k_B T := \lambda_1$$
$$\chi_{IJ} := \lambda_{IJ}^{\mathrm{epi}}$$
$$\chi_d^{\mathrm{dist}} := \lambda_d^{\mathrm{dist}}$$

We arrive at the following probability distribution:

$$\pi^{\mathrm{ME}}(\vec{r}) = \frac{e^{-\beta U^{\mathrm{ME}}(\vec{r})}}{\int d\vec{r} e^{-\beta U^{\mathrm{ME}}(\vec{r})}}$$

In our work, the resulting maximum entropy energy functional is:

$$U^{\mathrm{ME}}(\vec{r}) = U^{\mathrm{b}}(\vec{r}) + \sum_{c \in \mathrm{grid}} \sum_{\substack{I \geq J \\ I,J \in \mathrm{labels}}} \chi_{IJ} \frac{\Delta^3}{V_p} \phi_{I,c} \phi_{J,c} + \sum_{c \in \mathrm{grid}} \sum_{d=1}^{m} \chi_d^{\mathrm{dist}} \frac{\Delta^3}{V_p} \phi_{d,c}$$

#### S2.2.5 ME Optimization Procedure

For the generic observable $\nu_i(\vec{r})$ (e.g. $\nu_i(\vec{r}) = \psi_{:,I}^T H^{\mathrm{sim}} \psi_{:,J}$) we wish to determine the parameters $\lambda_i$ (e.g. $\chi_{IJ}$) contained in the potential energy:

$$U^{\mathrm{ME}}(\vec{r}; \vec{\lambda}) = U^{\mathrm{b}}(\vec{r}) + \sum_{i=1}^{n} \lambda_i \nu_i(\vec{r})$$

such that the expectation values of each observable coincides with its experimental value:

$$\langle \nu_i \rangle := \frac{1}{Z(\vec{\lambda})} \int e^{-\beta U^{\mathrm{ME}}(\vec{r}, \vec{\lambda})} \nu_i(\vec{r}) d\vec{r} = \nu_i^{\mathrm{exp}}$$

where $Z(\vec{\lambda}) = \int e^{-\beta U^{\mathrm{ME}}(\vec{r}, \vec{\lambda})} d\vec{r}$ is the partition function.
We can define a convex objective function $\theta$:

$$\theta(\vec{\lambda}) = \ln[Z(\vec{\lambda})] + \beta \sum_{i=1}^{n} \lambda_i \nu_i^{\mathrm{exp}}$$

The partial derivatives of the target function are:

$$g_i(\vec{\lambda}) = \frac{\partial \theta}{\partial \lambda_i} = -\beta \langle \nu_i \rangle_{\vec{\lambda}} + \beta \nu_i^{\text{exp}}$$

The Hessian is:

$$B_{ij} = \frac{\partial^2 \theta}{\partial \lambda_i \partial \lambda_j} = \beta^2 (\langle \nu_i \nu_j \rangle_{\vec{\lambda}} - \langle \nu_i \rangle_{\vec{\lambda}} \langle \nu_j \rangle_{\vec{\lambda}})$$

To find the optimal set of parameters, $\vec{\lambda}$, we numerically minimize the target function $\theta$ using Newton's method:

$$\lambda^{t+1} = \lambda^t - \gamma B^{-1}(\lambda^t) g(\lambda^t)$$

where $t$ is the iteration number and $\gamma$ is a dampening parameter.

We use $\gamma = 0.25$ for the first two iterations and $\gamma = 1$ for all subsequent iterations.

We define convergence when the normalized magnitude of the gradient is less than a convergence criterion, $\epsilon$:

$$\frac{|g(\vec{\lambda})|}{|\beta \vec{\nu}^{\text{exp}}|} < \epsilon$$

where $|\vec{x}|$ denotes the $l^2$-norm of $\vec{x}$

## S2.3 Neural Network Details

### S2.3.1 Message Passing Architecture

Our message passing architecture is adapted from the graph attention network proposed by Brody *et al.* [4]. Compared to Brody *et al.*, we include the edge features during the message passing step in addition to when calculating the attention coefficients.

$$X_i^{\text{new}} = \alpha_{i,i} \Theta_1 X_i + \sum_{j \in \mathcal{N}(i)} \alpha_{i,j} \Theta_2 [X_j \parallel E_{ij}] \tag{11}$$

where $X_i^{\text{new}}$ is the updated node embedding for node $i$, $\Theta_1$ and $\Theta_2$ are learnable weight matrices, $\mathcal{N}(i)$ is the set of neighbors of node $i$, $\parallel$ denotes concatenation, $E_{ij}$ is the edge features for the edge between node $i$ and $j$, and $\alpha_{i,j}$ is an attention coefficient defined as in Brody *et al.*:

$$\alpha_{i,j} = \frac{\exp\big(a^T \text{LeakyReLU}(\Theta[X_i \parallel X_j \parallel E_{ij}])\big)}{\sum_{k \in \mathcal{N}(i) \cup \{i\}} \exp\big(a^T \text{LeakyReLU}(\Theta[X_i \parallel X_j \parallel E_{ij}])\big)} \tag{12}$$

where $a$ and $\Theta$ are learnable weight matrices.

### S2.3.2 Neural Network Training

All neural networks are implemented and trained in PyTorch [21] using the PyTorch Geometric library [9]. We train/test on a synthetic dataset of $N = 10000$ samples generated as described in the main text. We train on 9000 samples and test on the remaining 1000.

We use the Adam optimizer with an initial learning rate (lr) of $10^{-4}$ and other parameters set to their defaults. We train for 40 epochs at lr=$10^{-4}$ followed by another 20 epochs with lr=$10^{-5}$.

### S2.3.3 Ablation Study

To assess the impact of variants of our data generation and training procedure, we performed an ablation study (Table A). We include all four metrics discussed in the main text in Table A: SCC, HiC-Spector, Corr PC1($\tilde{H}$), and RMSE($\tilde{H}$). For simplicity, we only discuss the SCC here.

*Baseline* refers to the GNN as described in the main text. All results in the main text use the Baseline GNN.

*No log-transform in loss* is a GNN trained using the mean-squared error between $\mathcal{U}$ and $\hat{\mathcal{U}}$ instead of between $\mathcal{U}^\dagger$ and $\hat{\mathcal{U}}^\dagger$. Training the GNN in this way noticeably decreases the SCC (0.573 vs 0.725), motivating our choice of defining the loss in the log space.

*50 kb resolution* uses a contact map at 50 kb instead of 100 kb resolution to define the graph structure. While this variant achieves lower MSE loss on the simulated validation set (0.313 vs 0.406), the SCC on the experimental validation set is slightly lower (0.650 vs 0.725). This result suggests that the GNN may be overfitting to the simulated contact maps when using the finer 50kb resolution.

*Without SignNet* omits the SignNet step described in the main text, where the entire GNN architecture is run twice, once with node features defined by positive eigenvectors and once by negative eigenvectors. We consider two variations. One where the eigenvectors are still included as a node feature, but their sign is unaccounted for - *Without SignNet (with eigenvectors)*. This variant performs substantially worse than the baseline (SCC 0.462 vs 0.725). And a second variation where the eigenvectors are omitted as well - *Without SignNet (without eigenvectors)*. Instead, the node features are initialized as a single constant value. Interestingly, this variant performs better than *Without SignNet (with eigenvectors)*, where the eigenvectors are included naively (SCC 0.647 vs 0.462).

*Without* $\text{mean}(\text{diagonal}(H, |i-j|))$ *in* $E_{ij}$ omits $\text{mean}(\text{diagonal}(H, |i-j|))$ from the edge features. This variant performs substantially worse (SCC 0.457 vs 0.725). Intuitively, we believe this feature to be important for the GNN to incorporate information about the contact probability scaling of the input contact map.

*Original message passing layer from [4]* uses the message passing layer as defined in Brody et al., instead of the modification we propose in Eq. (11). This variant performs dramatically worse (SCC -0.003 vs 0.725), demonstrating that incorporating edge features into the message passing step directly (not just when computation attention coefficients) is an essential modification.

## S2.4 Data Generation Details

### S2.4.1 Motivation

A major challenge in our problem setting is obtaining sufficient training data. To train the GNN in a supervised manner, we require a large dataset of contact maps, $H$, and the corresponding parameters, $\mathcal{U}$. There are two challenges to acquiring such a dataset. First, the number of existing experimental Hi-C contact maps is quite small compared to the scale of data needed to train modern neural networks. For instance, the ENCODE database contains only 195 different Hi-C experiments [18, 30]. Second, we do not know the corresponding polymer model parameters for these experimental contact maps, and estimating them via the maximum entropy approach would be computationally demanding. To address these challenges, we use synthetic data instead. The generalization properties and performance of the resulting trained neural network depend heavily on how the synthetic data is generated.

We seek to generate a training dataset of $(\mathcal{U}, H)$ pairs. Given a synthetic parameter matrix, $\mathcal{U}^s$, we can use our polymer model to produce the corresponding simulated contact map, $H^{\text{sim}}$. The challenge is to generate many $\mathcal{U}^s$ that are both biologically realistic and sufficiently diverse to allow the trained network to generalize well. We wish to leverage existing experimental data in combination with maximum entropy optimization solutions to strategically generate synthetic parameters. By using the maximum entropy parameter estimates to guide how we choose $\mathcal{U}^{\text{syn}}$, we can create realistic synthetic parameters. We expect these realistic parameters to yield simulated contact maps that resemble the experimental contact maps.

### S2.4.2 Method

First, we obtain a set of experimental contact maps at 50 kb resolution, $H^{(\ell)}$ for $\ell = 1, \ldots, 33$ corresponding to 25.6 Mb regions of the odd chromosomes of the IMR90 cell line. Each contact map contains $m = 512$ bins (i.e., rows/cols). For each $H^{(\ell)}$, we use the polymer model with the maximum entropy approach to estimate $\hat{L}^{\mathrm{ME}(\ell)}$ and $\hat{T}^{\mathrm{ME}(\ell)}$ matrices from Eq. (3). Define $\hat{\mathcal{U}}^{\mathrm{ME}(\ell)} := \hat{L}^{\mathrm{ME}(\ell)} + \hat{T}^{\mathrm{ME}(\ell)}$. We will use these maximum entropy energy matrices to guide the generation of synthetic data.

We generate 10,000 synthetic interaction parameter matrices, $\mathcal{U}^{(s)} \in R^{m \times m}$ for $s = 1, \ldots, 10000$ using the procedure in Algorithm F. In Algorithm F, we first generate $L^{(s)}$ using Algorithm G. Next, we generate $T^{(s)}$ as equal to the T matrix from a random maximum entropy simulation, $\hat{T}^{\mathrm{ME}(\ell_r)}$. We find that this simple procedure for defining $T^{(s)}$ is sufficient; the generative procedure for $L^{(s)}$ in Algorithm G helps generate diverse simulated contact maps.

To facilitate generating random $L^{(s)}$ matrices with Algorithm G, we first fit a distribution to the eigenvalues of $\hat{L}^{\mathrm{ME}(1)}, \ldots, \hat{L}^{\mathrm{ME}(33)}$. For each $\hat{L}^{\mathrm{ME}(\ell)}$, we compute the rank 10 eigendecomposition as $\hat{L}^{\mathrm{ME}(\ell)} = Q^{\mathrm{ME}(\ell)}\Lambda^{(\ell)}(Q^{\mathrm{ME}(\ell)})^T$ where $Q^{\mathrm{ME}(\ell)} \in \mathbb{R}^{m \times 10}$ contains the eigenvectors of $\hat{L}^{\mathrm{ME}(\ell)}$ and $\Lambda^{(\ell)} = \mathrm{diag}(\lambda_1^{(\ell)}, \ldots, \lambda_{10}^{(\ell)})$ is a diagonal matrix containing the eigenvalues of $\hat{L}^{\mathrm{ME}(\ell)}$. Note that $\hat{L}^{\mathrm{ME}(\ell)}$ is rank 10 by construction (Section S2.1.5), so this eigendecomposition is exact. Next, we fit a probability distribution, $p(\lambda_j)$ for $j = 1, \ldots, 10$, by kernel density estimation (KDE) over $\lambda_j^{(1)}, \ldots, \lambda_j^{(33)}$. For the KDE, we use a Gaussian kernel with bandwidth 10 using the implementation in Scikit-learn [22]. When fitting the KDE, we ignore outliers as defined by 1.5 times the interquartile range.

In Algorithm G, we define $L^{(s)}$ based on its eigendecomposition. First, we generate random eigenvalues, $\lambda_j^{(s)}$, by drawing from the probability distributions $p(\lambda_j)$ (lines 1-2). Next, we define the eigenvectors, $Q^{(s)}$, as equal to the eigenvectors from a random maximum entropy simulation, $Q^{\mathrm{ME}(\ell_q)}$ (line 3). In principle, we could design a generative model to generate random eigenvectors. However, we find using the maximum entropy eigenvectors without modification to yield sufficient results.

As an alternative to the approach in Algorithm G, we could generate $L^{(s)}$ by generating synthetic bead labels, $\Psi \in \mathbb{R}^{m \times k}$, and Flory-Huggin's parameters, $\chi \in \mathbb{R}^{k \times k}$ (Section S2.1, Eq. (6)). Note that $L := (\Psi\chi\Psi^T + (\Psi\chi\Psi^T)^T)/2$. In our approach, we only need to generate $k$ eigenvalues. To generate a synthetic $\chi$ would require $\frac{k(k+1)}{2}$ values ($\chi$ is symmetric). Further, the ordering of the bead labels in $\Psi$ is ambiguous. Consider that any column permutation of $\Psi$ is equally valid given the appropriate permutation of $\chi$. In our approach, the eigendecomposition naturally orders the eigenvectors by their eigenvalue, which makes generating synthetic eigenvalues more straightforward.

**Algorithm F** $\mathcal{U}^{(s)} \leftarrow \mathrm{Generate}\mathcal{U}$

$L^{(s)} \leftarrow \mathrm{GenerateL}()$
$T^{(s)} \leftarrow \hat{T}^{\mathrm{ME}(\ell_r)}$ for a random choice of $\ell_r \sim U\{1, \ldots, 33\}$
$\mathcal{U}^{(s)} \leftarrow L^{(s)} + T^{(s)}$
Return $\mathcal{U}^{(s)}$

**Algorithm G** $L^{(s)} \leftarrow \mathrm{GenerateL}$

**Given** $p(\lambda_j)$, KDE probability distributions for $j$th eigenvalue of $\hat{L}^{\mathrm{ME}}$
$Q^{\mathrm{ME}(1)}, \ldots, Q^{\mathrm{ME}(33)}$, eigenvectors of $\hat{L}^{\mathrm{ME}(1)}, \ldots, \hat{L}^{\mathrm{ME}(33)}$

1: Sample $\lambda_j^{(s)} \sim p(\lambda_j)$ for $j = 1, \ldots, 10$
2: $\Lambda^{(s)} \leftarrow \mathrm{diag}(\lambda_1^{(s)}, \ldots, \lambda_{10}^{(s)})$
3: $Q^{(s)} \leftarrow Q^{\mathrm{ME}(\ell_q)}$ for a random choice of $\ell_q \sim U\{1, \ldots, 33\}$ ▷ where $U$ is the uniform distribution
4: $L^{(s)} \leftarrow Q^{(s)}\Lambda^{(s)}(Q^{(s)})^T$
5: Return $L^{(s)}$

# S3 Robustness to Matrix Balancing Techniques

Matrix balancing techniques are a common pre-processing/normalization strategy used on experimental Hi-C data. These techniques 'balance' the contact map by ensuring that all rows/columns sum to a constant value. Matrix balancing has been shown to remove technical biases in experimental Hi-C data [11, 25]. Two popular matrix balancing tools used in the literature are ICE (Iterative Correction and Eigenvector decomposition) [11] and Knight-Ruiz Matrix Balancing [15].

To show that the performance of our GNN approach is unaffected by these common pre-processing tools, we evaluate our approach on KR and ICE pre-processed contact maps Fig. E. We download a raw contact map, perform matrix balancing, and then normalize the contact map as described in the main text. For these results, we have not retrained the GNN; it is trained on simulated data based on raw contact maps. Nonetheless, there is no significant difference in SCC when evaluated on matrix-balanced contact maps (Fig. E). This result suggests that the GNN has learned general properties of Hi-C data allowing it to generalize to matrix-balanced contact maps as well. This may be because matrix balancing has a relatively subtle effect on the resulting contact map. We do not expect the GNN to be compatibly with arbitrary pre-processing choices. Similarly, there is no significant difference in SCC for the maximum entropy approach.

We use the KR implementation from `https://github.com/ay-lab/HiCKRy` and the ICE implementation from `https://github.com/hiclib/iced`.

# S4 Robustness to Hi-C Data Quality

To demonstrate that the GNN is robust to the quality of the experimental data, we simulate the same genomic region using target contact maps with different numbers of total contacts. Contact maps with greater total contacts are of higher quality simply due to the law of large numbers. In our simulation, we can increase the number of total contacts by running longer simulations and sampling more structures. In the experiment, the total number of contacts is equal to the number of sequencing read-pairs (i.e., sequencing read depth) used to generate the contact map. Deeper sequencing runs will increase the number of total contacts.

To systematically achieve an *in silico* contact map with a desired number of total contacts, we randomly sample contacts with probability proportional to the contact probability from a high-quality experimental contact map until we reach the desired number of total contacts. We define the total contacts as the number of contacts in the target genomic region (as compared to genome-wide). Fig. FA-B shows the resulting *in silico* contact maps and corresponding simulated contact maps for a 25.6 Mb region of IMR90 Chr2 at $10^5$, $10^6$, and $10^7$ total contacts. The original experiment had $\sim 1.7 \times 10^7$ contacts in this region.

We performed this sampling procedure for ten different IMR90 contact maps to achieve *in silico* contact maps with total contacts between $10^4$ and $10^8$. Across the range of total contacts, the maximum entropy approach outperforms the GNN approach based on SCC (Fig. FC). For both approaches, the SCC plateaus for read depths greater than $10^6$ but decays relatively rapidly for lower read depths. When using the HiC-Spector metric, the GNN and maximum entropy approaches perform comparably for total contacts between $10^6$ and $10^8$ (Fig. FD). For total contacts less than $10^6$, the GNN approach's performance on the HiC-Spector metric degrades faster than the maximum entropy approach's performance. Note that the simulated contact maps on which the GNN was trained typically contain $10^6$-$10^7$ total contacts. As a result, it is unsurprising that the GNN performance deteriorates below a read depth of $10^6$.

Future work will be needed to extend the GNN to be successful on very sparse Hi-C data, as is common in the single-cell Hi-C setting. Single-cell Hi-C experiments typically yield $10^4$ to $10^6$ total contacts *per cell*. Assuming contacts are distributed uniformly across the genome, the 25.6 Mb regions here would only have $10^2$ to $10^4$ total contacts.

# S5 Relationship to Existing Machine Learning Methods

The graph attention architecture we use [4] is an example of a graph transformer. Similar to traditional transformer architectures, graph transformers rely on attention mechanisms [31] as their core building block. Notably, AlphaFold is a transformer architecture that has been remarkably successful in the protein structure prediction domain [1, 13, 16].

Since chromatin and proteins are both biological polymers, one might wonder if we could adapt AlphaFold to predict chromatin structure given appropriate inputs. Our approach has two key advantages over an AlphaFold-esque approach in the chromatin structure prediction setting. First, our approach can obtain accurate structure estimates with far less experimental training data. This is essential for chromatin structure prediction since we don't have large-scale experimental databases such as those available for protein structure [2]. Second, by using our polymer model, we can map an experimental contact map to a *distribution* of candidate structures rather than a *single* static structure, better reflecting the dynamic nature of the molecules. Effectively, our entire approach can be regarded as a physics-informed generative model for structures instead of a point estimate of a single structure, reflecting both (a) uncertainty in structure estimates and (b) the dynamic nature of chromatin.

# S6 Role of Machine Learning in Molecular Simulations

In computational chemistry and biology, coarse-grained particle-based simulation models are essential for studying the structural properties of polymers and large biomolecules. There are two basic frameworks for constructing coarse-grained models, referred to as 'bottom-up' and 'top-down' [20]. In the bottom-up framework, a coarse-grained model is parametrized to approximate an underlying fine-resolution model. Machine learning is commonly used in the bottom-up framework to learn effective coarse-grained potentials [10, 8].

On the other hand, the top-down framework does not require an underlying fine-resolution model. In the top-down framework, a model is constructed with user-defined energy functional consisting of unknown parameters that must be estimated from experimental data [20]. Our approach falls into this category. In the context of machine learning, top-down simulations can be considered an inverse problem where experimental data is observed, but the parameters of the underlying energy functional are unobserved. There has been relatively little work developing machine learning approaches to parametrize coarse-grained models in the top-down framework [29]. We hope that our approach spurs further development for machine learning tools in the top-down setting.

# S7 Metrics for Comparing Contact Maps

The first metric we consider is the stratum-adjusted correlation coefficient (SCC), which is a weighted average of the Pearson correlation between pairs of off-diagonals of two Hi-C contact maps [33]. To compute the SCC, we wrote our own implementation which operates on dense matrices. The SCC requires two user-defined parameters: a smoothing parameter, $h$, and the maximum range of interactions to consider (i.e., the maximum off-diagonal to consider, $K$). We consider only contacts within the range of 0-5 Mb, as in the original method from Yang et al. [33]. This corresponds to the first $K = 100$ off-diagonals for our resolution of 50 kb. For smoothing, we use a 2D mean filter with a span size of $h = 5$. We choose $h = 5$ because our resolution of 50 kb is closest to the recommendation of using $h = 5$ for 40 kb resolution from Yang et al. We show the effect of varying the filter span size, $h$, (while $K = 100$) in Fig. H.

As an alternative metric, we consider the Hi-C Spector score [32]. Given two Hi-C contact maps, the Hi-C Spector score first converts each contact map into a graph Laplacian matrix. The Hi-C Spector score is a scaled sum of the Euclidean norm between the first $\kappa$ Laplacian eigenvectors. The metric is scaled to the range $[0, 1]$, where 1 is better. We compute the HiC-Spector score using the implementation provided in `https://github.com/gersteinlab/HiC-spector` with the first $\kappa = 10$ Laplacian eigenvectors. Empirically,

past work has found the Hi-C Spector score to be more robust to the Hi-C data quality (ie., sequencing read depth) than the SCC [34].

As seen in Table 1 and described in the main text, the maximum entropy approach outperforms the GNN approach based upon the SCC (the Hi-C Spector score is comparable). Despite this, we find that the maximum entropy simulated contact maps often have noticeable visual 'imperfections'. In Fig. I, we show four example contact maps where the maximum entropy approach tends to overestimate experimental contact frequencies in some portions of the contact map. The corresponding genomic-distance normalized contact maps are shown in Fig. J. Note that these examples are not curated; they are the first four contact maps in our experimental test set - all from chromosome 2 of the IMR90 cell line. The significance of these imperfections is unclear, and therefore we include two additional metrics, Corr PC1($\tilde{H}$) and RMSE($\tilde{H}$).

Corr PC1($\tilde{H}$) and RMSE($\tilde{H}$) are both measures of the global similarity of genomic-distance normalized contact maps. In contrast, both SCC and HiC-Spector focus primarily on contacts near the main diagonal. By design, the SCC only focuses on contacts within 5 Mb. In addition, the SCC gives higher weight to contacts near the main diagonal. HiC-Spector also focuses on contacts near the main diagonal since it treats the contact map as a graph and compares Laplacian eigenvectors. The primary signal in this graph Laplacian will be the main diagonal of the contact map.

Depending on the application, it may be preferable to focus on the main diagonal (where the majority of contacts are located) or to focus on the compartmentalization pattern. Visually, the GNN approach seems to do better at reproducing long-range compartmentalization patterns (Fig. I, Fig. J). This is supported by the GNN's better performance on the Corr PC1($\tilde{H}$) and RMSE($\tilde{H}$) metrics (Table 1).

The origin of this performance difference may be a consequence of differences in the GNN loss function and maximum entropy optimization objective. We train the GNN with mean-squared error loss between the ground truth and estimated $\mathcal{U}$ matrices. As a consequence, the GNN gives equal weight to all entries in $\mathcal{U}$. This may explain why the GNN performs best on global measures of contact map similarity. On the other hand, the maximum entropy optimization objective seeks to constrain the average contact frequencies between beads of type $I$ and $J$ to match between the simulation and experiment (see Section S2.2 for details of the maximum entropy approach). Since the majority of contacts are located near the main diagonal, the maximum entropy approach prioritizes performance near the main diagonal.

# S8 References


[1] Mehmet Akdel, Douglas E.V. Pires, Eduard Porta Pardo, Jürgen Jänes, Arthur O. Zalevsky, Bálint Mészáros, Patrick Bryant, Lydia L. Good, Roman A. Laskowski, Gabriele Pozzati, Aditi Shenoy, Wensi Zhu, Petras Kundrotas, Victoria Ruiz Serra, Carlos H.M. Rodrigues, Alistair S. Dunham, David Burke, Neera Borkakoti, Sameer Velankar, Adam Frost, Jérôme Basquin, Kresten Lindorff-Larsen, Alex Bateman, Andrey V. Kajava, Alfonso Valencia, Sergey Ovchinnikov, Janani Durairaj, David B. Ascher, Janet M. Thornton, Norman E. Davey, Amelie Stein, Arne Elofsson, Tristan I. Croll, and Pedro Beltrao. A structural biology community assessment of AlphaFold2 applications. *Nature Structural and Molecular Biology*, 29(11):1056–1067, 11 2022.

[2] Helen M Berman, John Westbrook, Zukang Feng, Gary Gilliland, T N Bhat, Helge Weissig, Ilya N Shindyalov, and Philip E Bourne. The Protein Data Bank. *Nucleic Acids Research*, 28(1):235–242, 2000.

[3] Bogdan Bintu, Leslie J. Mateo, Jun Han Su, Nicholas A. Sinnott-Armstrong, Mirae Parker, Seon Kinrot, Kei Yamaya, Alistair N. Boettiger, and Xiaowei Zhuang. Super-resolution chromatin tracing reveals domains and cooperative interactions in single cells. *Science*, 362(6413):1–8, 10 2018.

[4] Shaked Brody, Uri Alon, and Eran Yahav. How Attentive are Graph Attention Networks? *arXiv*, 2022.

[5] Franois A. Detcheverry, Darin Q. Pike, Umang Nagpal, Paul F. Nealey, and Juan J. De Pablo. Theoretically informed coarse grain simulations of block copolymer melts: Method and applications. *Soft Matter*, 5(24):4858–4865, 2009.

[6] François A. Detcheverry, Huiman Kang, Kostas Ch Daoulas, Marcus Müller, Paul F. Nealey, and Juan J. De Pablo. Monte Carlo simulations of a coarse grain model for block copolymers and nanocomposites. *Macromolecules*, 41(13):4989–5001, 7 2008.

[7] Michele Di Pierro, Bin Zhang, Erez Lieberman Aiden, Peter G. Wolynes, and José N. Onuchic. Transferable model for chromosome architecture. *Proceedings of the National Academy of Sciences of the United States of America*, 113(43):12168–12173, 10 2016.

[8] Aleksander E.P. Durumeric, Nicholas E. Charron, Clark Templeton, Félix Musil, Klara Bonneau, Aldo S. Pasos-Trejo, Yaoyi Chen, Atharva Kelkar, Frank Noé, and Cecilia Clementi. Machine learned coarse-grained protein force-fields: Are we there yet? *Current Opinion in Structural Biology*, 79, 4 2023.

[9] Matthias Fey and Jan Eric Lenssen. Fast Graph Representation Learning with PyTorch Geometric. *arXiv*, pages 1–9, 3 2019.

[10] Paraskevi Gkeka, Gabriel Stoltz, Amir Barati Farimani, Zineb Belkacemi, Michele Ceriotti, John D. Chodera, Aaron R. Dinner, Andrew L. Ferguson, Jean Bernard Maillet, Hervé Minoux, Christine Peter, Fabio Pietrucci, Ana Silveira, Alexandre Tkatchenko, Zofia Trstanova, Rafal Wiewiora, and Tony Lelièvre. Machine Learning Force Fields and Coarse-Grained Variables in Molecular Dynamics: Application to Materials and Biological Systems. *Journal of Chemical Theory and Computation*, 16(8):4757–4775, 8 2020.

[11] Maxim Imakaev, Geoffrey Fudenberg, Rachel Patton McCord, Natalia Naumova, Anton Goloborodko, Bryan R. Lajoie, Job Dekker, and Leonid A. Mirny. Iterative correction of Hi-C data reveals hallmarks of chromosome organization. *Nature Methods*, 9(10):999–1003, 10 2012.

[12] E T Jaynes. Information Theory and Statistical Mechanics. *Physical Review*, 106(4):620–630, 1957.

[13] John Jumper, Richard Evans, Alexander Pritzel, Tim Green, Michael Figurnov, Olaf Ronneberger, Kathryn Tunyasuvunakool, Russ Bates, Augustin Žídek, Anna Potapenko, Alex Bridgland, Clemens Meyer, Simon A.A. Kohl, Andrew J. Ballard, Andrew Cowie, Bernardino Romera-Paredes, Stanislav Nikolov, Rishub Jain, Jonas Adler, Trevor Back, Stig Petersen, David Reiman, Ellen Clancy, Michal Zielinski, Martin Steinegger, Michalina Pacholska, Tamas Berghammer, Sebastian Bodenstein, David Silver, Oriol Vinyals, Andrew W. Senior, Koray Kavukcuoglu, Pushmeet Kohli, and Demis Hassabis. Highly accurate protein structure prediction with AlphaFold. *Nature*, 596(7873):583–589, 8 2021.

[14] A. Khalil, J. L. Grant, L. B. Caddle, E. Atzema, K. D. Mills, and A. Arneodo. Chromosome territories have a highly nonspherical morphology and nonrandom positioning. *Chromosome Research*, 15(7):899–916, 2007.

[15] Philip A. Knight and Daniel Ruiz. A fast algorithm for matrix balancing. *IMA Journal of Numerical Analysis*, 33(3):1029–1047, 7 2013.

[16] Andriy Kryshtafovych, Torsten Schwede, Maya Topf, Krzysztof Fidelis, and John Moult. Critical assessment of methods of protein structure prediction (CASP)—Round XIV. *Proteins: Structure, Function and Bioinformatics*, 89(12):1607–1617, 12 2021.

[17] Xingcheng Lin, Yifeng Qi, Andrew P. Latham, and Bin Zhang. Multiscale modeling of genome organization with maximum entropy optimization. *Journal of Chemical Physics*, 155(1):1–24, 2021.

[18] Yunhai Luo, Benjamin C. Hitz, Idan Gabdank, Jason A. Hilton, Meenakshi S. Kagda, Bonita Lam, Zachary Myers, Paul Sud, Jennifer Jou, Khine Lin, Ulugbek K. Baymuradov, Keenan Graham, Casey Litton, Stuart R. Miyasato, J. Seth Strattan, Otto Jolanki, Jin Wook Lee, Forrest Y. Tanaka, Philip Adenekan, Emma O'Neill, and J. Michael Cherry. New developments on the Encyclopedia of DNA Elements (ENCODE) data portal. *Nucleic Acids Research*, 48(D1):D882–D889, 1 2020.

[19] Quinn MacPherson, Bruno Beltran, and Andrew J. Spakowitz. Bottom–up modeling of chromatin segregation due to epigenetic modifications. *Proceedings of the National Academy of Sciences of the United States of America*, 115(50):12739–12744, 12 2018.

[20] W. G. Noid. Perspective: Coarse-grained models for biomolecular systems. *Journal of Chemical Physics*, 139(9):1–25, 9 2013.

[21] Adam Paszke, Sam Gross, Francisco Massa, Adam Lerer, James Bradbury, Gregory Chanan, Trevor Killeen, Zeming Lin, Natalia Gimelshein, Luca Antiga, Alban Desmaison, Andreas Köpf, Edward Yang, Zach DeVito, Martin Raison, Alykhan Tejani, Sasank Chilamkurthy, Benoit Steiner, Lu Fang, Junjie Bai, and Soumith Chintala. PyTorch: An Imperative Style, High-Performance Deep Learning Library. *arXiv*, pages 1–12, 12 2019.

[22] Fabian Pedregosa, Gaël Varoquaux, Alexandre Gramfort, Vincent Michel, Bertrand Thirion, Olivier Grisel, Mathieu Blondel, Peter Prettenhofer, Ron Weiss, Vincent Dubourg, Jake Vanderplas, Alexandre Passos, David Cournapeau, Matthieu Brucher, Matthieu Perrot, and Édouard Duchesnay. Scikit-learn: Machine Learning in Python. *Journal of Machine Learning Research*, 12(85):2825–2830, 2011.

[23] Darin Q. Pike, Frațois A. Detcheverry, Marcus Müller, and Juan J. De Pablo. Theoretically informed coarse grain simulations of polymeric systems. *Journal of Chemical Physics*, 131(8):1–10, 2009.

[24] Yifeng Qi and Bin Zhang. Predicting three-dimensional genome organization with chromatin states. *PLoS Computational Biology*, 15(6):1–21, 6 2019.

[25] Suhas S.P. Rao, Miriam H. Huntley, Neva C. Durand, Elena K. Stamenova, Ivan D. Bochkov, James T. Robinson, Adrian L. Sanborn, Ido Machol, Arina D. Omer, Eric S. Lander, and Erez Lieberman Aiden. A 3D map of the human genome at kilobase resolution reveals principles of chromatin looping. *Cell*, 159(7):1665–1680, 12 2014.

[26] Michael Rubinstein and Ralph H. Colby. *Polymer Physics*. Oxford University Press, 2003.

[27] Guang Shi and D. Thirumalai. A maximum-entropy model to predict 3D structural ensembles of chromatin from pairwise distances with applications to interphase chromosomes and structural variants. *Nature Communications*, 14(1):1150, 3 2023.

[28] Jun Han Su, Pu Zheng, Seon S. Kinrot, Bogdan Bintu, and Xiaowei Zhuang. Genome-Scale Imaging of the 3D Organization and Transcriptional Activity of Chromatin. *Cell*, 182(6):1641–1659, 9 2020.

[29] Stephan Thaler and Julija Zavadlav. Learning neural network potentials from experimental data via Differentiable Trajectory Reweighting. *Nature Communications*, 12(1), 12 2021.

[30] The ENCODE Project Consortium. An integrated encyclopedia of DNA elements in the human genome. *Nature*, 489(7414):57–74, 9 2012.

[31] Ashish Vaswani, Noam Shazeer, Niki Parmar, Jakob Uszkoreit, Llion Jones, Aidan N. Gomez, Lukasz Kaiser, and Illia Polosukhin. Attention Is All You Need. *arXiv*, pages 1–15, 6 2017.

[32] Koon Kiu Yan, Galip Gürkan Yardlmcl, Chengfei Yan, William S. Noble, and Mark Gerstein. HiC-spector: A matrix library for spectral and reproducibility analysis of Hi-C contact maps. *Bioinformatics*, 33(14):2199–2201, 7 2017.

[33] Tao Yang, Feipeng Zhang, Galip Gürkan Yardımci, Fan Song, Ross C. Hardison, William Stafford Noble, Feng Yue, and Qunhua Li. HiCRep: assessing the reproducibility of Hi-C data using a stratum-adjusted correlation coefficient. *Genome Research*, 27(11):1939–1949, 11 2017.

[34] Galip Gürkan Yardımcı, Hakan Ozadam, Michael E.G. Sauria, Oana Ursu, Koon Kiu Yan, Tao Yang, Abhijit Chakraborty, Arya Kaul, Bryan R. Lajoie, Fan Song, Ye Zhan, Ferhat Ay, Mark Gerstein, Anshul Kundaje, Qunhua Li, James Taylor, Feng Yue, Job Dekker, and William S. Noble. Measuring the reproducibility and quality of Hi-C data. *Genome Biology*, 20(1):1–19, 3 2019.

[35] Bin Zhang and Peter G. Wolynes. Topology, structures, and energy landscapes of human chromosomes. *Proceedings of the National Academy of Sciences of the United States of America*, 112(19):6062–6067, 5 2015.

# S9 Supplementary Figures

| Method | MSE | SCC | HiC-Spector | Corr PC1($\tilde{H}$) | RMSE($\tilde{H}$) |
|---|---|---|---|---|---|
| Baseline | 0.406 | 0.725 | 0.533 | 0.913 | 0.677 |
| No log-transform in loss | NA | 0.573 | 0.468 | 0.620 | 0.805 |
| 50 kb resolution | 0.313 | 0.650 | 0.503 | 0.825 | 0.682 |
| Without SignNet (with eigenvectors) | 0.501 | 0.462 | 0.421 | 0.827 | 0.867 |
| Without SignNet (without eigenvectors) | 0.484 | 0.647 | 0.468 | 0.809 | 0.707 |
| Without mean(diagonal($H, \|i-j\|$)) in $E_{ij}$ | 0.470 | 0.457 | 0.432 | 0.738 | 0.802 |
| Original message passing layer from [4] | 1.784 | -0.003 | 0.379 | 0.204 | 0.843 |

Table A: Neural network ablation results. See Section S2.3.3 for a description of each method. All results in the body of the paper correspond to the baseline method. MSE is the average mean-squared error on the validation set of 1000 simulated contact maps and synthetic parameters. The MSE is reported after log-transformation of $\mathcal{U}$ to $\mathcal{U}^{\dagger}$ as described in the main text. SCC, HiC-Spector, Corr PC1($\tilde{H}$), and RMSE($\tilde{H}$) are computed between each experimental contact map and the contact map simulated using the GNN-predicted parameters, averaged over 33 experimental contact maps from the odd chromosomes of the IMR90 cell line.

| Method | SCC | HiC-Spector | Corr PC1($\tilde{H}$) | RMSE($\tilde{H}$) | Simulation Time |
|---|---|---|---|---|---|
| GM12878, HMEC, HUVEC, HAP1 | | | | | |
| GNN | 0.71 ± 0.01 | 0.54 ± 0.01 | 0.85 ± 0.01 | 0.70 ± 0.01 | 11.4 ± 0.1 |
| Maximum Entropy | 0.82 ± 0.00 | 0.52 ± 0.01 | 0.79 ± 0.01 | 1.07 ± 0.01 | 75.3 ± 2.0 |
| GM12878 | | | | | |
| GNN | 0.74 ± 0.01 | 0.55 ± 0.02 | 0.86 ± 0.02 | 0.49 ± 0.01 | 9.5 ± 0.1 |
| Maximum Entropy | 0.83 ± 0.01 | 0.52 ± 0.02 | 0.84 ± 0.02 | 0.92 ± 0.03 | 72.8 ± 4.1 |
| HMEC | | | | | |
| GNN | 0.66 ± 0.02 | 0.53 ± 0.03 | 0.82 ± 0.03 | 0.7 ± 0.01 | 13.5 ± 0.2 |
| Maximum Entropy | 0.83 ± 0.01 | 0.55 ± 0.03 | 0.76 ± 0.04 | 0.95 ± 0.02 | 77.9 ± 5.3 |
| HUVEC | | | | | |
| GNN | 0.66 ± 0.02 | 0.53 ± 0.03 | 0.82 ± 0.03 | 0.7 ± 0.01 | 13.5 ± 0.2 |
| Maximum Entropy | 0.83 ± 0.01 | 0.55 ± 0.03 | 0.76 ± 0.04 | 0.95 ± 0.02 | 77.9 ± 5.3 |
| HAP1 | | | | | |
| GNN | 0.7 ± 0.01 | 0.56 ± 0.02 | 0.83 ± 0.03 | 0.76 ± 0.01 | 11.0 ± 0.3 |
| Maximum Entropy | 0.82 ± 0.01 | 0.54 ± 0.02 | 0.79 ± 0.02 | 1.18 ± 0.03 | 84.3 ± 4.5 |
| CH12.LX | | | | | |
| GNN | 0.78 ± 0.01 | 0.42 ± 0.02 | 0.8 ± 0.04 | 0.84 ± 0.02 | 9.2 ± 0.2 |
| Maximum Entropy | 0.81 ± 0.01 | 0.38 ± 0.02 | 0.85 ± 0.01 | 1.29 ± 0.02 | 77.0 ± 4.6 |

Table B: Average results for experimental contact maps from even chromosomes of GM12878, HMEC, HUVEC, HAP1, and CH12.LX (GNN was trained only using the IMR90 cell line). Metrics are defined as in Table 1. Maximum entropy optimization was performed with a convergence criterion of $\epsilon = 10^{-2}$. All values are mean ± standard error.

| Cell Line | Genome Version | Repository | File |
|---|---|---|---|
| IMR90 | hg19 | Juicebox | hiseq/imr90/in-situ/combined.hic |
| GM12878 | hg19 | Juicebox | hiseq/gm12878/in-situ/combined.hic |
| HMEC | hg19 | Juicebox | hiseq/hmec/in-situ/combined.hic |
| HUVEC | hg19 | Juicebox | hiseq/huvec/in-situ/combined.hic |
| HAP1 | hg19 | Juicebox | hiseq/hap1/in-situ/combined.hic |
| CH12.LX | mm9 | Juicebox | hiseq/ch12-lx-b-lymphoblasts/in-situ/combined.hic |
| IMR90 | hg38 | ENCODE | ENCSR345VTI/ENCFF281ILS.hic |

Table C: Experimental data sources used in this manuscript. The Juicebox repository can be found at https://www.aidenlab.org/data.html. The ENCODE repository can be found at https://www.encodeproject.org/.

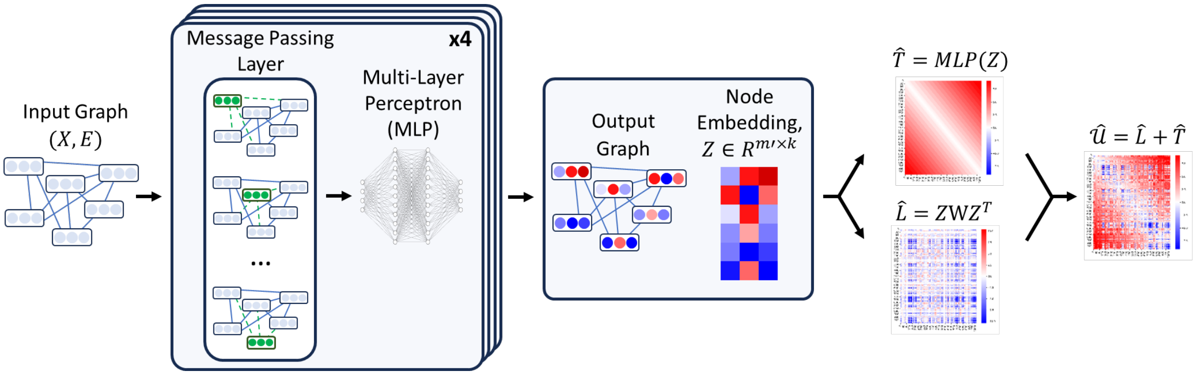


Figure A: Schematic overview of GNN architecture. We define a graph (not to scale), node features ($X$), and edge features ($E$) based on a Hi-C contact map. The core graph neural network comprises a message passing layer and a multi-layer perception (MLP). After four iterations, the GNN yields a node embedding, $Z$. The node embedding is used to predict $\hat{L}$ via a bilinear function and $\hat{T}$ via another MLP. $\hat{L}$ and $\hat{T}$ are summed (element-wise) to yield $\hat{\mathcal{U}}$.

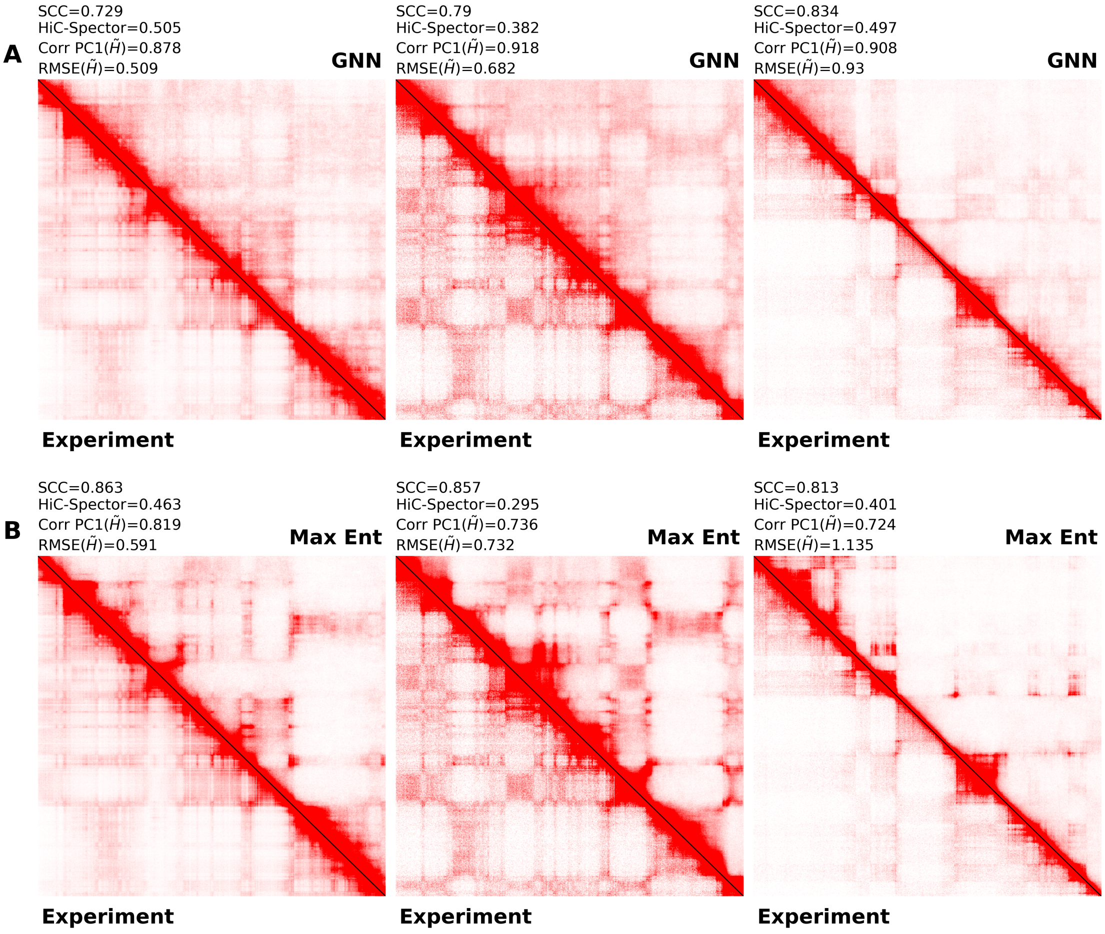


Figure B: Comparison of GNN and maximum entropy simulated contact maps vs. experimental contact map of Chr2:5.1-30.7 Mb from three cell lines. *Left:* GM12878 human lymphoblastoid cell line. *Middle:* HMEC human mammary epithelial cell line. *Right:* HUVEC human umbilical vein endothelial cell line. The lower triangle shows the experiment. The color bar is the same for all subfigures. (A) The upper triangle shows the GNN simulation. (B) The upper triangle shows the maximum entropy simulation.

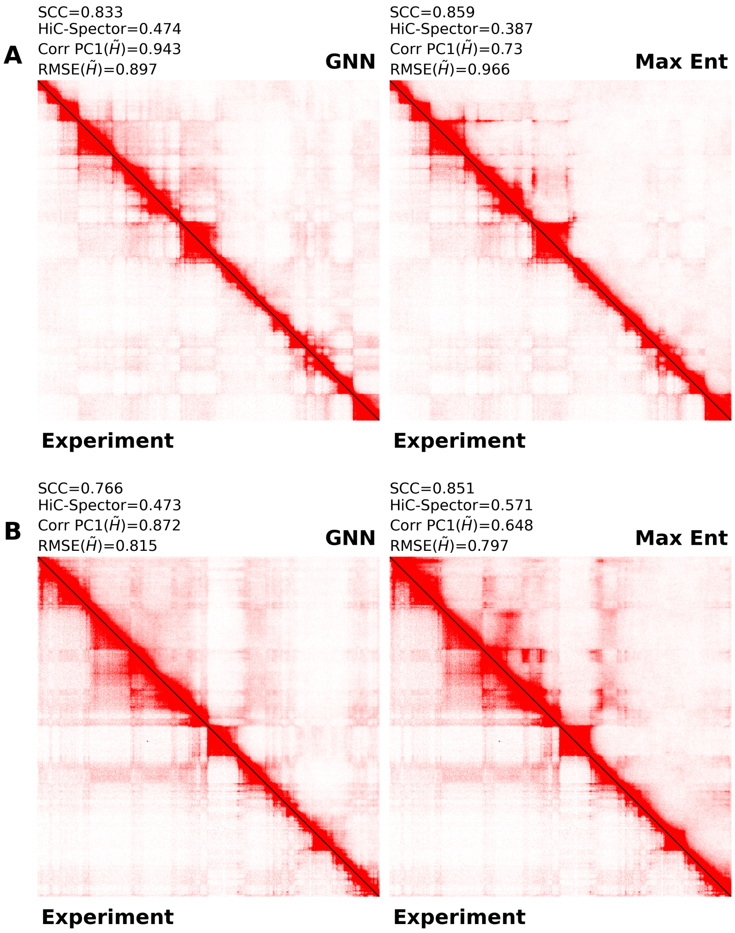


Figure C: Contact maps corresponding to distances analysis in Fig. 4. (A) IMR90 Chr2:10-25.6 Mb. (B) IMR90 Chr21:14-39.6 Mb. The lower triangle shows the experimental contact map. *Left:* The upper triangle shows the GNN simulated contact map. *Right:* The upper triangle shows the maximum entropy simulated contact map.

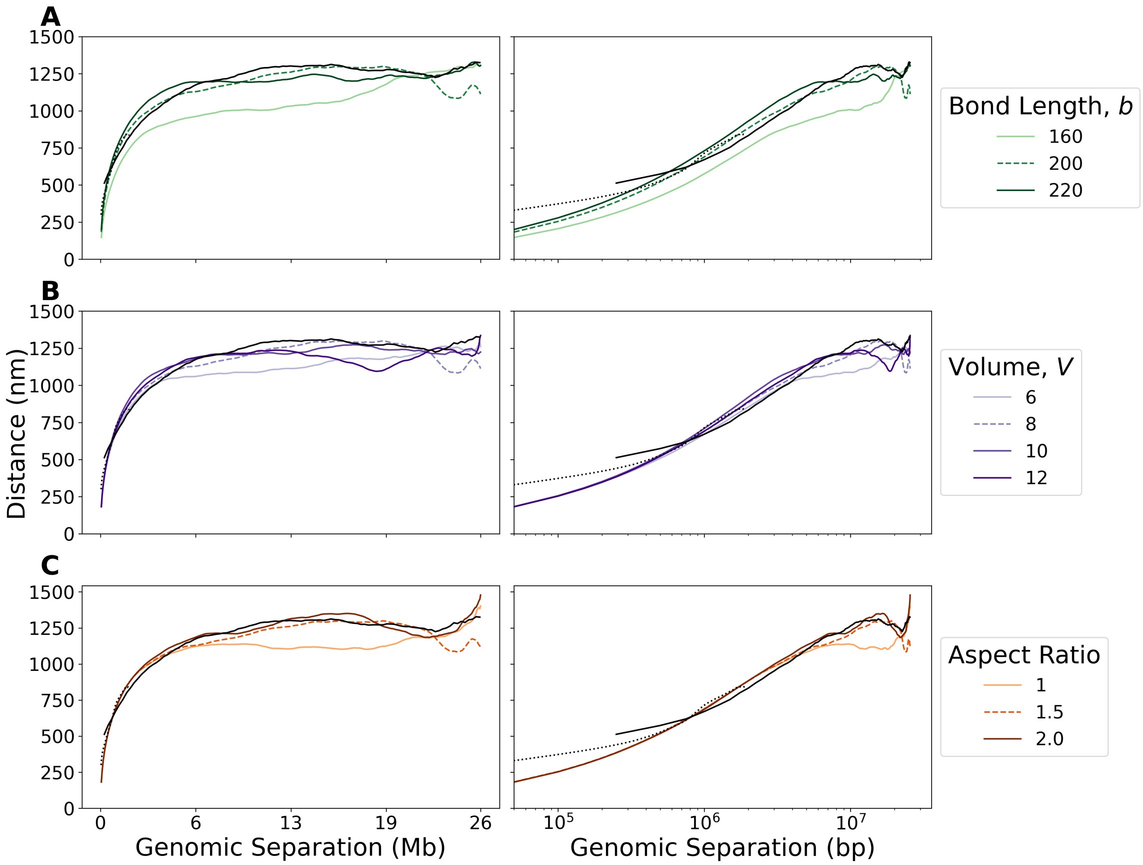


Figure D: Mean distance scaling as function of genomic separation. The vertical axis is the mean euclidean distance averaged over structures and averaged over pairs of loci at a given genomic separation. The two black curves are experimental distance scaling for IMR90 Chr2:10-25.6 Mb. The solid black curve is at 50 kb resolution from [28], and the dotted black curve is at 30 kb resolution from [3]. Colored curves are simulated distance scalings for maximum entropy optimized simulations. The dashed curve in each panel corresponds to the parameters used in the main text. *Left:* Linear scale x-axis *Right:* Log scale x-axis (A) Varying bond length, $b$, holding $V = 8\ \mu\text{m}^3$ and aspect ratio $= 1.5$ (B) Varying simulation volume, $V$, holding $b = 200$ nm$^3$ and aspect ratio $= 1.5$ (C) Varying aspect ratio holding $b = 200$ nm$^3$ and $V = 8\ \mu\text{m}^3$

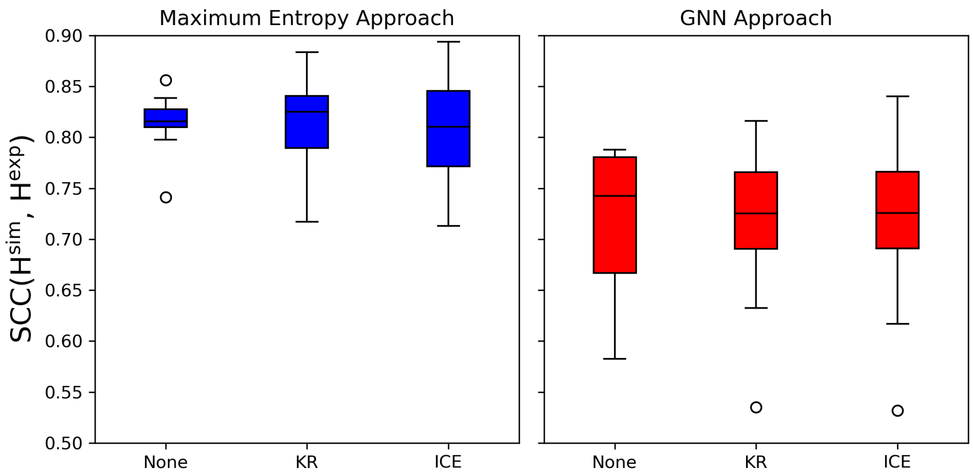


Figure E: Effect of pre-processing with Knight-Ruiz (KR) and ICE matrix balancing techniques on model performance. The vertical axis is the SCC between each experimental contact map and the simulated contact map for 33 experimental contact maps from the odd chromosomes of the IMR90 cell line. *Left*: Maximum entropy approach. *Right*: GNN Approach. Boxes represent the 25th and 75th quantiles, and the solid line indicates the median. Whiskers extend from the box by 1.5 times the interquartile range.

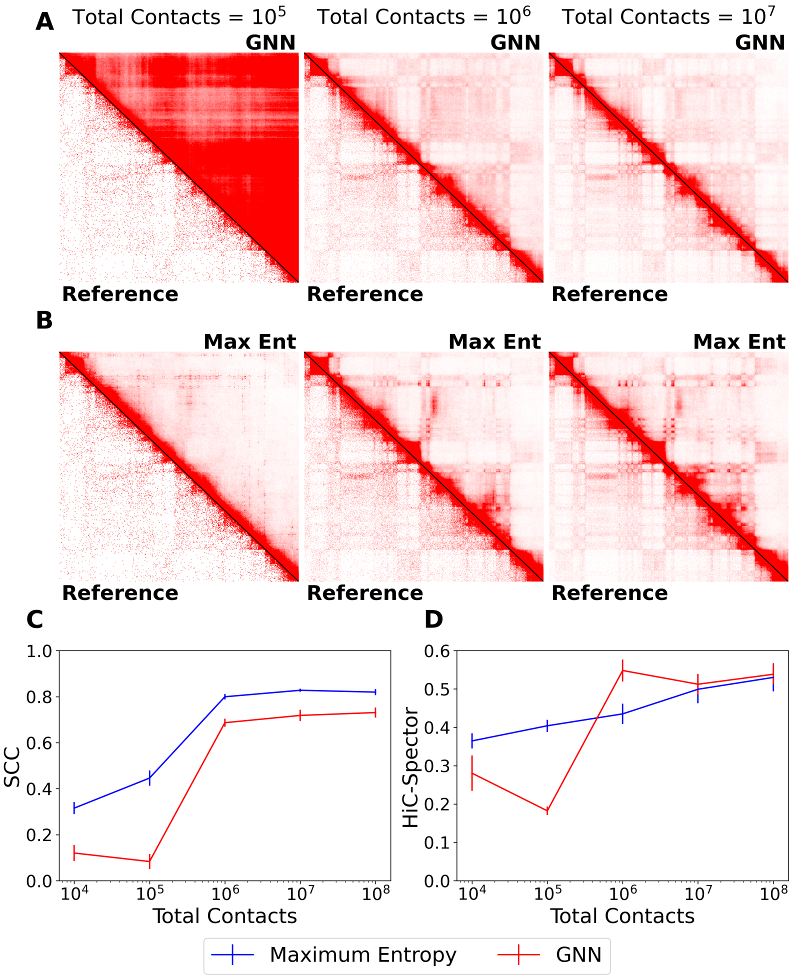


Figure F: Effect of contact map quality on GNN and maximum entropy performance. (A-B) Contact maps of IMR90 Chr2:149.75-175.35 Mb at different numbers of total contacts. *Left:* Total contacts of $10^5$. *Middle:* Total contacts of $10^6$. *Right:* Total contacts of $10^7$. The lower triangle shows the experimental contact map subsampled to the indicated total contacts. The color bar is the same for all subfigures. (A) The upper triangle is the GNN simulated contact map. (B) The upper triangle is the maximum entropy simulated contact map. (C-D) Average SCC (C) and HiC-Spector score (D) across ten IMR90 contact maps as a function of total contacts for GNN (red) and maximum entropy (blue) simulations. Error bar shows $\pm$ one standard deviation.

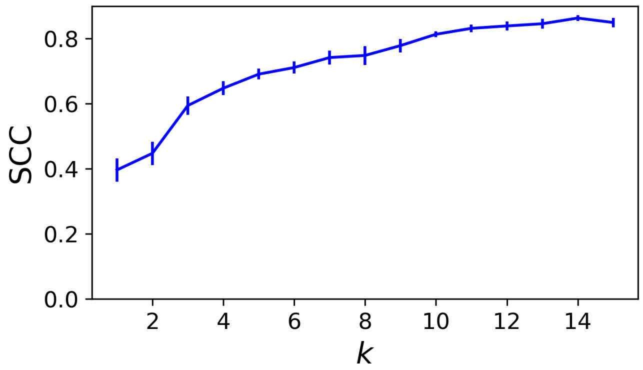


Figure G: Effect of the number of principal components, $k$, used in the maximum entropy approach on the SCC between experimental and simulated contact maps. SCC is the average SCC from 10 contact maps in the experimental validation set of contact maps from the odd chromosomes of the IMR90 cell line. Error bars show standard error.

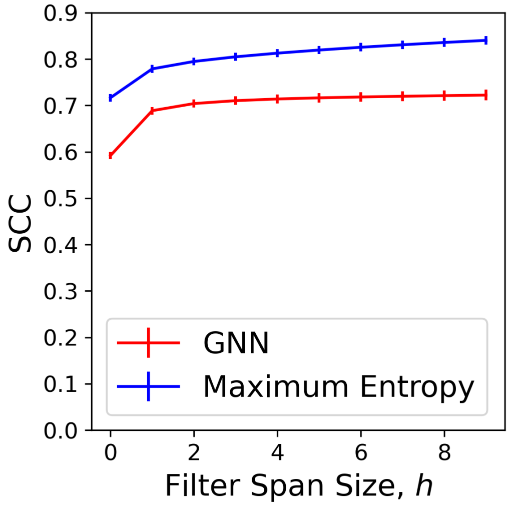


Figure H: Effect of the filter span size, $h$, when calculating SCC. See Section S7 for details. SCC is the average SCC from 40 contact maps in the experimental test set of contact maps from the even-chromosomes of the IMR90 cell line. SCC is reported for both the GNN approach (red) and maximum entropy approach (blue). Error bars show standard error.

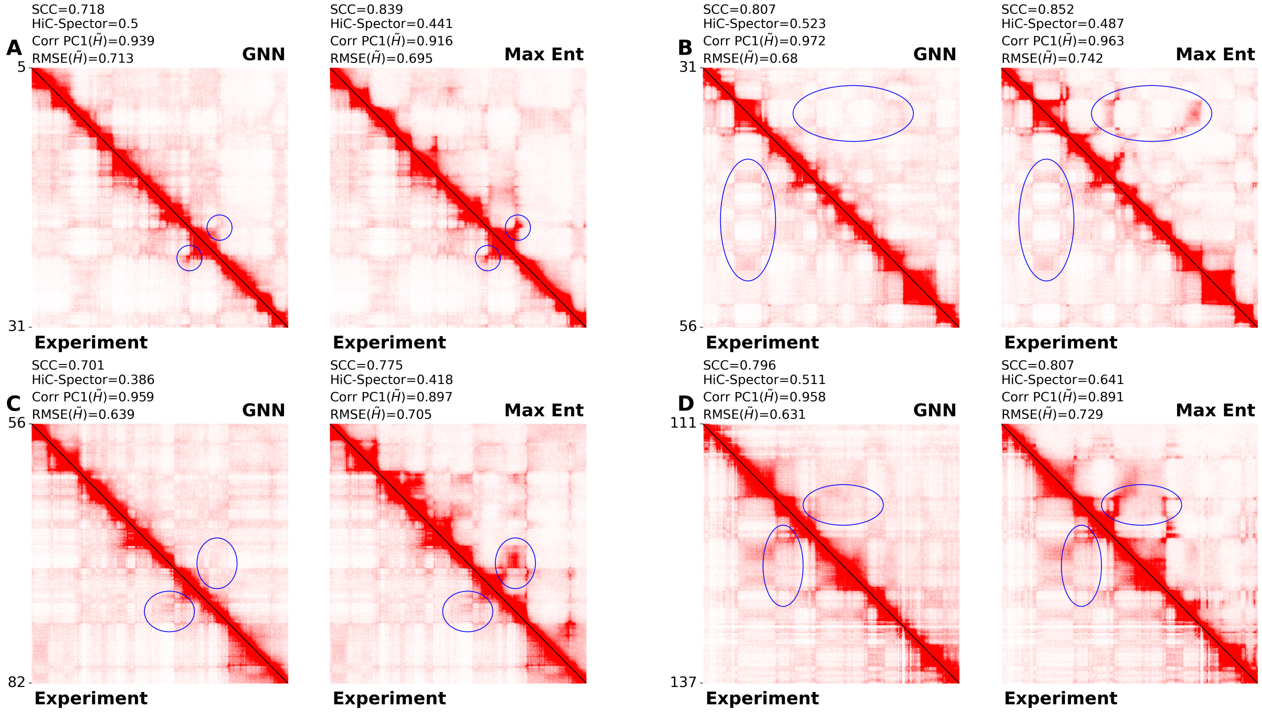


Figure I: Comparison of GNN and maximum entropy simulated contact maps vs. experimental contact maps. The experimental contact map is always shown in the lower triangle. *Left:* upper triangle is GNN. *Right:* upper triangle is maximum entropy. Blue ellipses highlight regions for which the maximum entropy simulation overestimates the experimental contact frequencies. (A) IMR90 Chr2:5.1-30.7 Mb. (B) IMR90 Chr2:30.7-56.3 Mb. (C) IMR90 Chr2:56.3-81.9 Mb. (D) IMR90 Chr2:111.3-136.9 Mb.

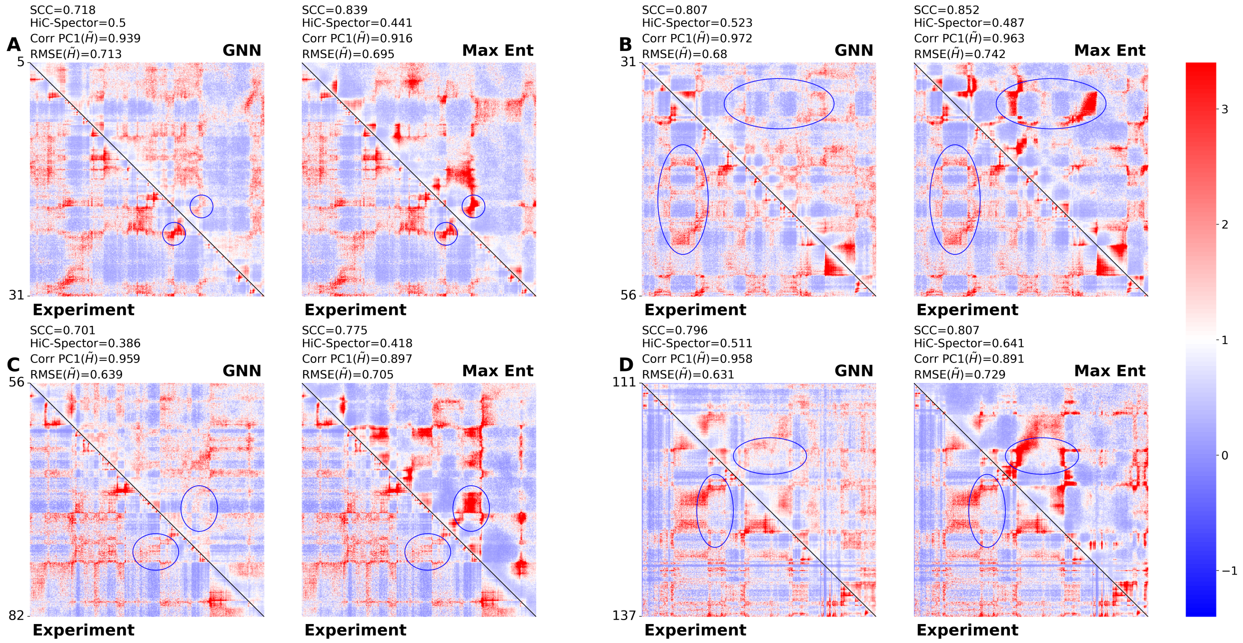


Figure J: Comparison of genomic-distance normalized contact maps as in Fig. I. The experimental contact map is always shown in the lower triangle. *Left:* upper triangle is GNN. *Right:* upper triangle is maximum entropy. Blue ellipses highlight regions for which the maximum entropy simulation overestimates the experimental contact frequencies. (A) IMR90 Chr2:5.1-30.7 Mb. (B) IMR90 Chr2:30.7-56.3 Mb. (C) IMR90 Chr2:56.3-81.9 Mb. (D) IMR90 Chr2:111.3-136.9 Mb.
